# Structural and Spectroscopic Properties of Isoconazole and Bifonazole—Experimental and Theoretical Studies

**DOI:** 10.3390/ijms24010520

**Published:** 2022-12-28

**Authors:** Beata Drabińska, Katarzyna Dettlaff, Tomasz Ratajczak, Kacper Kossakowski, Marcin K. Chmielewski, Judyta Cielecka-Piontek, Jacek Kujawski

**Affiliations:** 1 Chair and Department of Organic Chemistry, Faculty of Pharmacy, Poznan University of Medical Sciences, Grunwaldzka 6 Str., 60-780 Poznań, Poland; 2 Chair and Department of Pharmaceutical Chemistry, Faculty of Pharmacy, Poznan University of Medical Sciences, Grunwaldzka 6 Str., 60-780 Poznań, Poland; 3 Liquid Dosage Form Laboratory, Research and Development Department, Polfa Warszawa S.A, Karolkowa 22/24 Str., 01-207 Warsaw, Poland; 4 Centre of New Technologies, University of Warsaw, Banacha 2C Str., 02-097 Warsaw, Poland; 5 Biosynthesis Sp. z o.o., Rubież 46 Str., 61-612 Poznań, Poland; 6 Institute of Bioorganic Chemistry, Polish Academy of Sciences, Noskowskiego 12/14 Str., 61-704 Poznań, Poland; 7 Chair and Department of Pharmacognosy, Faculty of Pharmacy, Poznan University of Medical Sciences, Rokietnicka 3 Str., 60-780 Poznań, Poland

**Keywords:** density functional theory, natural bond orbital, nuclear magnetic resonance

## Abstract

The paper compares the experimental FT-IR, UV-vis, and ^1^H NMR spectra of isoconazole and bifonazole with the density functional theory (DFT) calculations using different functionals. The results were compared with previously reported data related to their analogue, posaconazole. The analysis of calculated IR spectra with use of CAM-B3LYP (isoconazole) or B3LYP (bifonazole) functionals shows good accordance with the experimental IR spectrum. The best compatibility between the experimental and theoretical UV spectra was observed with the use of B3LYP or wB97XD functionals for isoconazole or bifonazole, respectively. The reason for the difference in the UV-vis spectra of isoconazole and bifonazole was discussed based on linear response time-dependent DFT and natural bond orbital methods. The calculated ^1^H NMR spectrum shows that the DFT formalism, particularly the B3LYP functional, give an accurate description of the isoconazole and bifonazole chemical shifts.

## 1. Introduction

Azoles are the most widely used antifungal drugs, acting by inhibiting the lanosterol 14-α-demethylase, the fungal cytochrome P450 enzyme (CYP51A1), which is responsible for conversion of lanosterol to the ergosterol, major sterol agent in the fungal cell wall [1]. The structure of the wall is disrupted by changing permeability of membrane and leading to the cell death. Two classes of antifungal azoles are currently in clinical use. The first class is the imidazole group, where compounds consist of two-nitrogen azole ring, and the second is the triazole group, which includes compounds with three nitrogens in the azole ring. Nitrogen atoms in imidazole and triazole moiety are the key to binding via coordination with the heme iron of the enzyme. Moreover, changes in the lipophilic side chain of azoles can cause the differences in affinity of binding with enzyme due interaction with CYP51 apoprotein [2]. These molecular aspects lead to the search for new azole analogues for a more effective treatment of mycoses.

Isoconazole belongs to the *N*-substituted imidazole class of azoles with four chlorine atoms and is closely similar in structure to miconazole. It is used in pharmaceutical preparations as nitrate salts and is the active pharmaceutical ingredient in the topical drugs Travocort and Travogen (Bayer HealthCare/Intendis GmbH) [3]. Additionally, Travocort is a mixture of isoconazole and diflucortolone valerate, which as topical corticosteroid reduces the inflammation associated with fungal infection. Both drugs were specifically developed to treat superficial fungal skin diseases due to their favorable pharmacokinetics characteristic related with rapid penetration of human skin and broad spectrum of antimicrobial properties. Isoconazole has activity against microbials, such as dermatophytes, pathogenic yeast, filamentous fungi, moulds, and Gram-positive bacteria, e.g., *Corynebacterium Minutissimum* [3].

Bifonazole belongs to the *N*-substituted imidazole class of azoles and is structurally related to other drugs from this group, such as clotrimazole, econazole, and miconazole. It is available in various formulations for topical use, such as cream, gel, or powder, and should be used once daily, which is an advantage allowing increased compliance [4]. Bifonazole has a broad spectrum of activity against dermatophytes and yeasts and has also been shown to inhibit some Gram-positive bacteria, such as *Staphylococcus*, *Streptococcus*, and *Corynebacterium species*. It has confirmed efficiency in the treatment of superficial skin infections such as tinea corporis, tinea crusis, tinea manuum, tinea pedis interdigitalis, cutaneous candidiasis, pityriasis versicolor, and erythrasma [4]. The newest research suggests that bifonazole is potentially SARS-CoV-2 blocking agent because it restricts infection and replication of native SARS-CoV-2 [5].

Continuing our computational chemistry investigations on biologically active azoles [6,7,8], we focused our attention on experimental and theoretical analysis of spectroscopic properties of isoconazole and bifonazole. To the best of our knowledge, there are limited studies regarding spectral analysis of isoconazole and bifonazole. The reference data are limited to experimental studies of UV-Vis spectra [9,10,11], FT-IR spectra for bifonazole [12], and DFT (density functional theory and the B3LYP/6-311++G(d,p) approximation) computations regarding conformational analysis and quantum descriptors for two bifonazole derivatives [13]. Therefore, we compared results of the experimental FT-IR, UV–Vis, and ^1^H NMR spectra of bifonazole and isoconazole with calculated data by applying different basis sets and methods in the gaseous phase and using the conductor-like polarizable continuum (CPCM) solvation model to include the solvent influence on the chemical environment. The main goal of our investigations was to prove what basis sets or methods yield results in the best agreement with the experimental data. Although compounds have nitrogen atoms within their structure, the low abundance of the ^15^N isotope in comparison with the ^1^H isotope as well as the significant signal broadening due to the large quadrupole moment of N renders the nitrogen NMR spectroscopy impractical [14]. ^1^H NMR spectroscopy is broadly used in confirming the identity and purity of small molecule organic compounds. The ^13^C NMR methodology, on the other hand, finds application, e.g., with low-permeability investigations [15], analysis of modern materials such as coal samples [16], understanding reactive behaviors and mechanisms of oxygen carriers [17], and in the hydrodeoxygenation (HDO) process, being considered an efficient method to remove the oxygenated groups for further improving the quality of biooils [18]. Considering the low utility of ^13^C NMR spectroscopy for the investigation of interactions of small molecule organic compounds as well as the low natural abundance of ^13^C, we decided to use ^1^H NMR for the studies depicted in our previously published paper [19]. Moreover, we hoped to explain which conformation of the compounds is preferred from the standpoint of quantum chemistry. Such analysis might translate into a better understanding of the compounds’ structural features responsible for their biological action. Furthermore, we expected to provide a rational explanation for the striking difference in the UV spectrum of isoconazole and bifonazole. The above issues are worth examining more closely as the spectral analysis of these drugs involving DFT formalism and its comparison with the experimental data remain relatively undeveloped.

## 2. Results and Discussion

### 2.1. Geometry Optimisation

**Figure 1:**
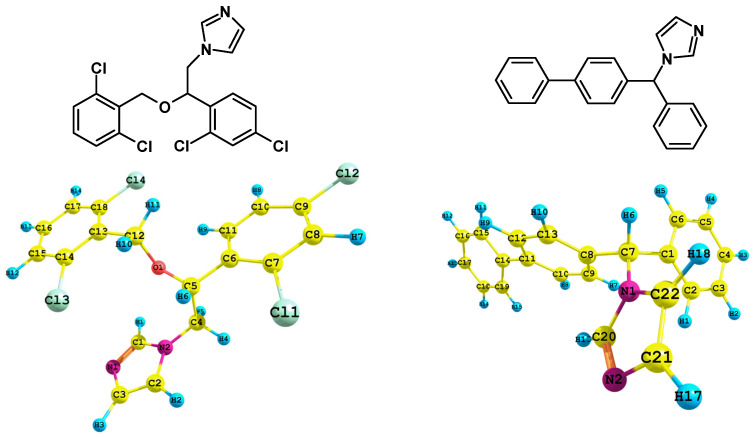
Formulas for isoconazole (**1**) and bifonazole (**2**) and their optimized geometry at the CAM-B3LYP/6-31G(d,p) (**1**, **left**) or B3LYP/6-31G(d,p) levels of theory (**2**, **right**).

Considering the initial geometries of the rotamers isoconazole **1** and bifonazole **2** (formulas are given in the Figure 1), the magnitude of the RMSD error for the geometry of remaining optimized rotamers was 0.43971 and 0.4301 (for rotamers optimized at the B3LYP/6-31G(d,p) level of theory), 0.4214 and 0.4537 (for rotamers optimized at the B3LYP/6-311++G(d,p) level of theory), 0.3495 and 0.4610 (CAM-B3LYP/6-31G(d,p)), 0.4418 and 0.5073 (PBE1PBE/6-31G(d,p)), 0.5528 and 0.4773 (M06L/6-31G(d,p)), 0.6811 and 0.5217 (M062X/6-31G(d,p)), 0.4426 and 0.4684 Å (APF/6-31G(d,p)), 1.7556 and 0.4770 Å (wB97XD/6-31G(d,p)), respectively. It is therefore shown that the smallest RMSD value for isoconazole **1** was obtained with respect to the CAM-B3LYP/6-31G(d,p) approximation. In contrast, the smallest RMSD value concerning the optimized rotamer geometry of bifonazole **2** was obtained using the B3LYP functional. Next, the molecular electrostatic potential (MEP) was determined by the CAM-B3LYP/6-311++G(2d,3p) or B3LYP/6-311++G(2d,3p) approaches for the selected rotamer of azole **1** or **2**, respectively, with geometry previously optimized in gaseous phase.

**Figure 2:**
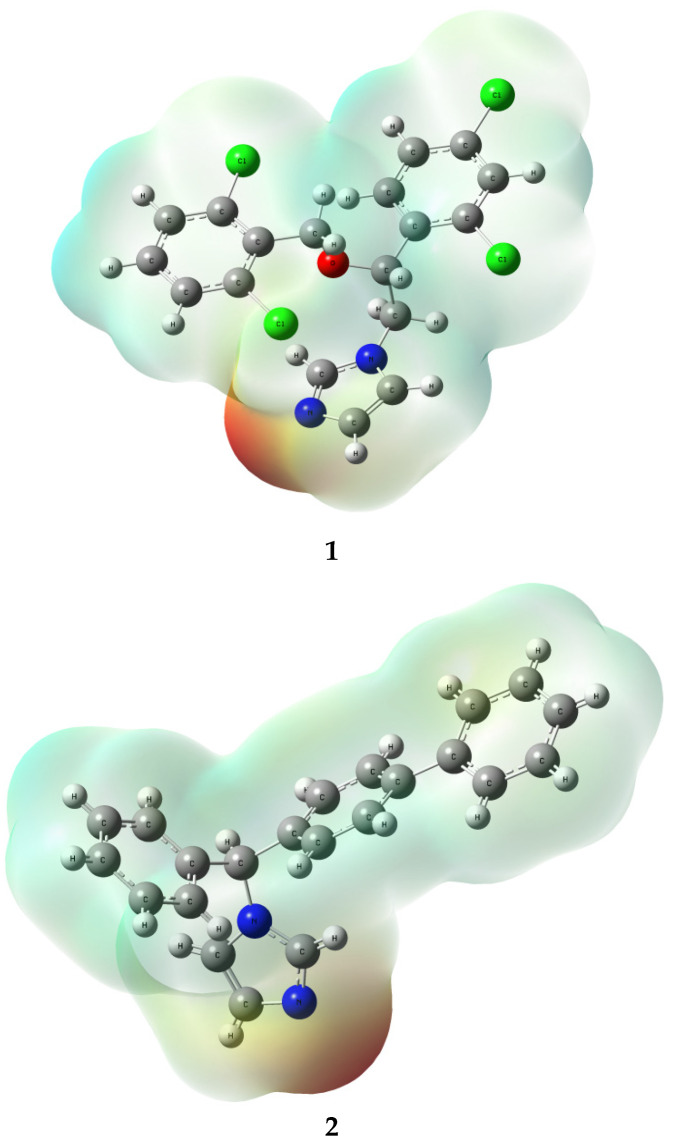
Electrostatic potential (ESP) map of compounds **1** (**up**) and **2** (**down**) calculated at the CAM-B3LYP/6-311++G(2d,3p)//CAM-B3LYP/6-31G(d,p) (**1**) or B3LYP/6-311++G(2d,3p)//B3LYP/6-31G(d,p) (**2**) levels of theory; gaseous phase; isovalue = 0.0004 a.u.; scale: red–blue from −6.243 × 10^−2^ to +6.243 × 10^−2^.

Regarding the analysis of MEP (Figure 2) for the charge distribution in the **1** and **2** optimized rotamers, we employed the keywords: “pop=full”, as well as the natural bond orbital (NBO, key-words: “pop=nbo”) as implemented in Gaussian software, and CHelpG (charges from electrostatic potential) procedure (key-word “pop=chelpg”). In the latter scheme, atomic charges are fitted to reproduce the molecular electrostatic potential at several points around the molecule.

Of all the nitrogen atoms within the structure of **1** and **2** (Table 1 and Table 2, respectively) the pyrrolic nitrogen’s atoms N1 (**2**) and N1 (**1**) are characterized by more positive charge, however the application of the NBO charges methodology indicated these atoms as the ones having the negative charge value. The N1 (**1**) or N2 (**2**) atoms were found to have the most negative charge value when the CAM-B3LYP functional and the CHelpG charges methodology were applied. In the case of the CHelpG charges methodology, all chloride atoms within the structure of isoconazole **1** are characterized by negative values of charges.

### 2.2. IR Analysis

**Figure 3:**
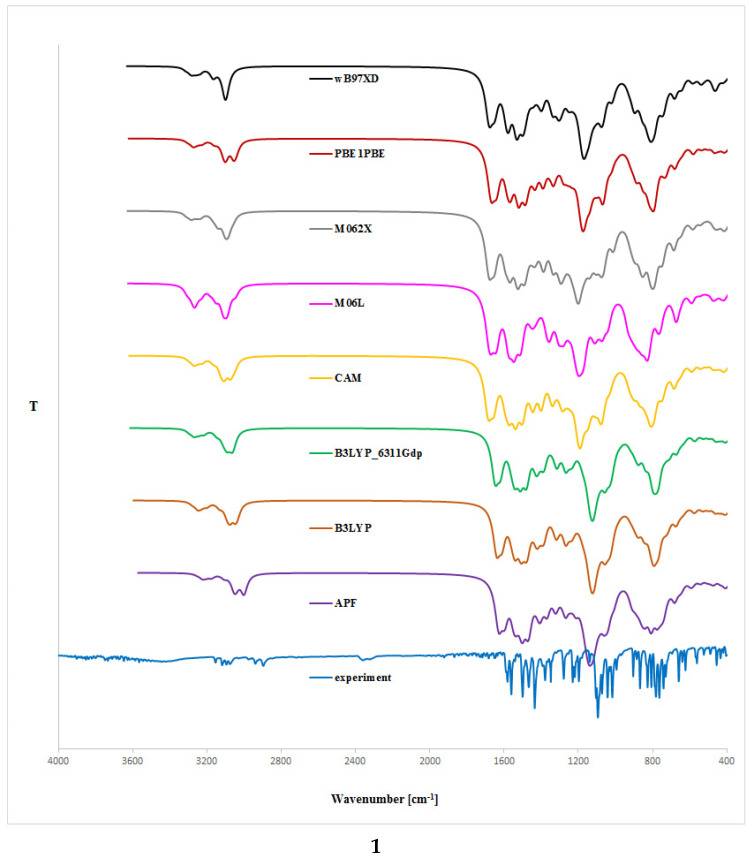

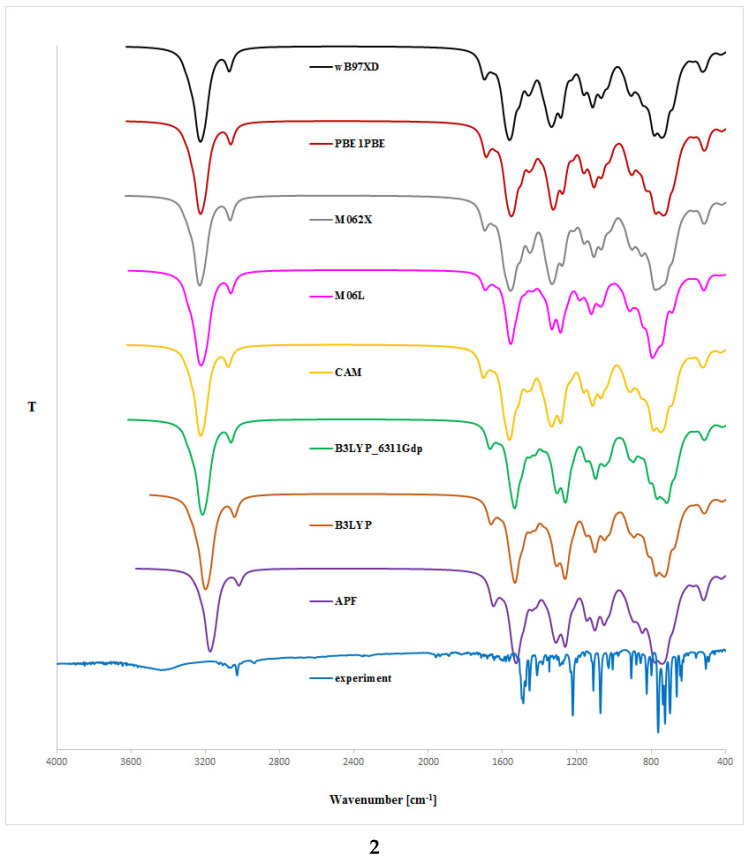
Expended experimental (EXP) and theoretical IR spectra (DFT formalism, gaseous phase) of isoconazole (**1**) or bifonazole (**2**); **B3LYP**—B3LYP/6-31G(d,p)//B3LYP/6-31G(d,p) approach, **APF**-APF/6-31G(d,p)//APF/6-31G(d,p) approach, **B3LYP_6311Gdp**-B3LYP/6-311++G(d,p)//B3LYP/6-311++G(d,p) approach, **CAM**-CAM-B3LYP/6-31G(d,p)//CAM-B3LYP/6-31G(d,p) approach, **M06L**-M06L/6-31G(d,p)//M06L/6-31G(d,p) approach, **M062X**-M062X/6-31G(d,p)//M062X/6-31G(d,p) approach, **PBE1PBE**-PBE1PBE/6-31G(d,p)//PBE1PBE/6-31G(d,p) approach, **wB97XD**-wB97XD/6-31G(d,p)//wB97XD/6-31G(d,p) approach.

The theoretical analysis of isoconazole (**1**) and bifonazole (**2**) IR spectrum was limited to the DFT formalism without correction term. We carried out the computations of **1** and **2** vibrational frequencies using the same level of theory as was used for the SCF optimization procedure and Grimme’s D3 empirical (GD3) dispersion model [20] (for rotamers optimized in gaseous phase using B3LYP, CAM-B3LYP, PB0, M06L, M062X, wB97XD functionals; Table 3 and Table 4), and the Petersson–Frisch dispersion model from the APFD functional (for rotamer optimized using APF functional) [21]. The resultant IR spectra are shown in Figure 3. Small differences between the experimental and calculated vibrational modes can be observed because the experimental results were obtained in solid phase whereas the theoretical calculations were carried out in gaseous phase.

From the spectra given in Figure 3, we can conclude that the use of the B3LYP/6-31G(d,p), CAM-B3LYP/6-31G(d,p), or even wB97XD/6-31G(d,p) and PBE1PBE/6-31G(d,p) approaches for the rotamers optimization gives the highest conformity of the theoretical IR bands with the experimental spectrum, particularly in the ca. 3100–2800 cm^−1^ and ca. 1750–500 cm^−1^ range. The utilization of the Grimme’s D3 empirical dispersion model leads to better consistency between the theoretical and experimental IR spectral values. Moreover, including the diffuse functions in B3LYP/6-311++G(d,p) approach did not lead to the theoretical IR spectrum being comparable with the experimental one (as is relatively more time-consuming in comparison with the B3LYP/6-31G(d,p) functional). In the computed spectra of **1** and **2**, the estimated υ and δ C-Cl, N=N, C-N (within the azole moiety), and γ C-H (related with benzene and azole rings) absorptions were in excellent accordance with the experimental and literature data [22]. To the best of our knowledge, in the literature, there are no data regarding IR spectrum simulation using DFT formalism for isoconazole **1**. It turned out that the use of the B3LYP, CAM-B3LYP, wB97XD, or PBE1PBE functional was comparatively more effective because these approaches generally afford results without significant errors. A similar conclusion cannot be drawn from the method involving other functionals used in our investigations.

### 2.3. UV-Vis Analysis

**Figure 4:**
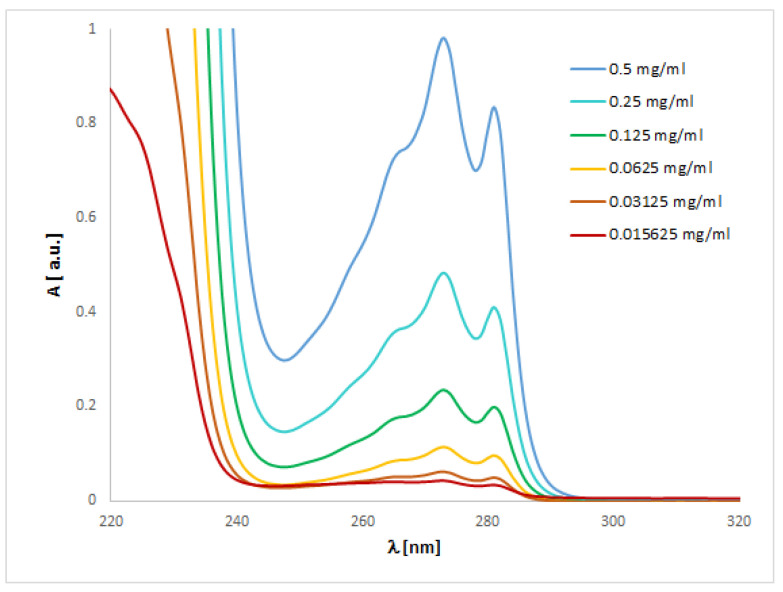
Experimental UV-vis spectrum of isoconazole **1** registered in methanol at various concentrations.

**Figure 5:**
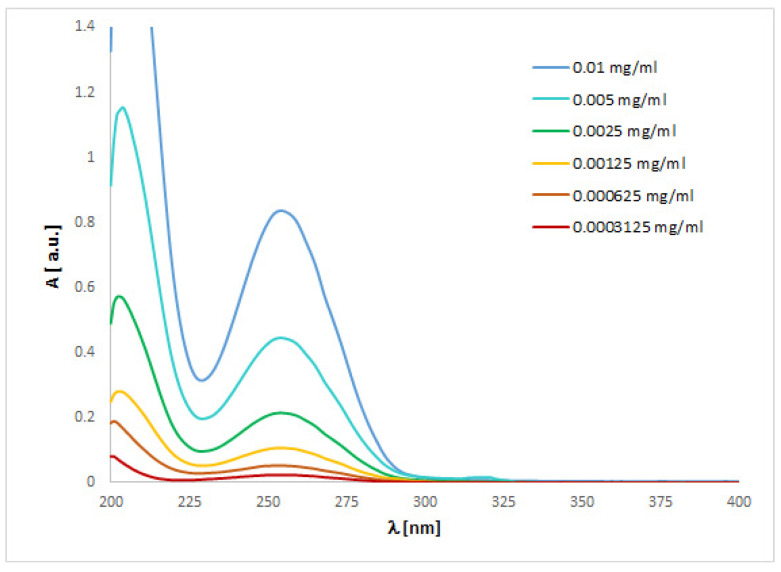
Experimental UV-vis spectrum of bifonazole **2** registered in methanol at various concentrations.

The UV spectra regarding the absorption bands of **1** and **2** are in accordance with the literature data [11]. The spectra (Figure 4 and Figure 5) display an absorption band at 273 (for **1**) or 254 nm (for **2**) nm, which did not change with the concentration used. However, other absorption bands of analytes **1** or **2** are observed at 219–229 (**1**) or 201–213 (**2**) nm and 281–283 (**1**) nm, which migrated as a function of concentration.

To match the experimental (Figure 4 and Figure 5) and theoretical UV-vis spectra of analytes **1** and **2** (Figures S1–S4, Supplementary Material), we optimized the molecules geometry and applied linear response time-dependent DFT (TDDFT) method for the calculations. The vertical excited states were calculated for each optimized rotamer of compounds **1** and **2** at the functional/6-311++G(2d,3p) level of theory in gas phase, as well as in methanol, chloroform, dichloromethane, and acetonitrile (CPCM solvation model).

**Figure 6:**
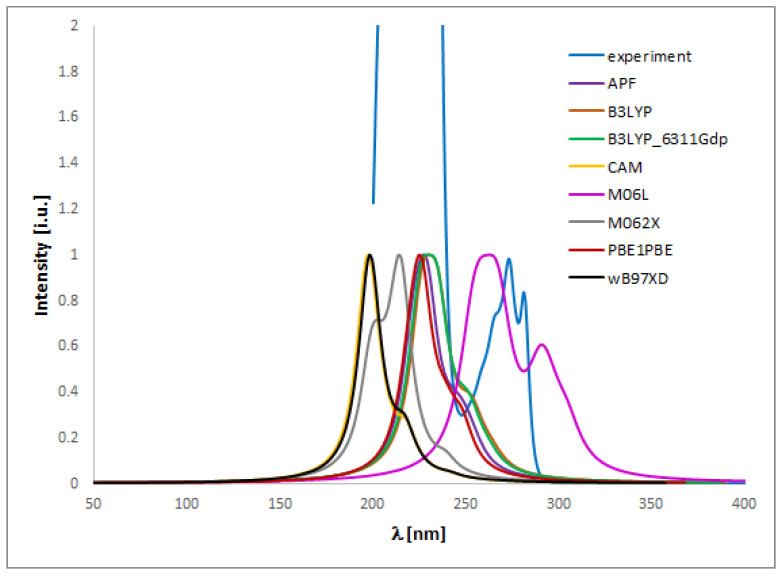
Experimental (experimental) and theoretical UV-vis spectra of isoconazole **1** registered in methanol and computed using CPCM solvation model (methanol as solvent); approximations: **APF**-APF/6-311++G(2d,3p)//APF/6-31G(d,p), **B3LYP**-B3LYP/6-311++G(2d,3p)//B3LYP/6-31G(d,p), **B3LYP_6311_Gdp-**B3LYP/6-311++G(2d,3p)//B3LYP/6-311++G(d,p), **CAM**-CAM-B3LYP/6-311++G(2d,3p)//CAM-B3LYP/6-31G(d,p), **M06L**-M06L/6-311++G(2d,3p)//M06L/6-31G(d,p), **M062X**-M062X/6-311++G(2d,3p)//M062X/6-31G(d,p), **PBE1PBE**-PBE1PBE/6-311++G(2d,3p)//PBE1PBE/6-31G(d,p).

**Figure 7:**
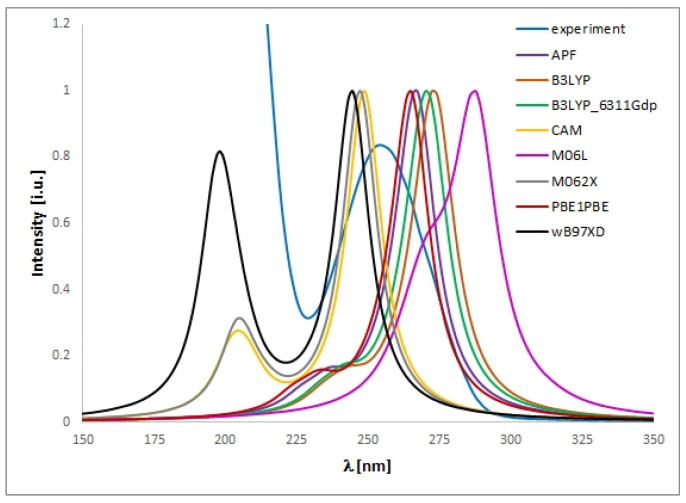
Experimental (experimental) and theoretical UV-vis spectra of bifonazole **2** registered in methanol and computed using CPCM solvation model (methanol as solvent); approximations: **APF**-APF/6-311++G(2d,3p)//APF/6-31G(d,p), **B3LYP**-B3LYP/6-311++G(2d,3p)//B3LYP/6-31G(d,p), **B3LYP_6311_Gdp-**B3LYP/6-311++G(2d,3p)//B3LYP/6-311++G(d,p), **CAM**-CAM-B3LYP/6-311++G(2d,3p)//CAM-B3LYP/6-31G(d,p), **M06L**-M06L/6-311++G(2d,3p)//M06L/6-31G(d,p), **M062X**-M062X/6-311++G(2d,3p)//M062X/6-31G(d,p), **PBE1PBE**-PBE1PBE/6-311++G(2d,3p)//PBE1PBE/6-31G(d,p).

In the case of isoconazole **1** (Figure 6), the highest correspondence to the experimental data, especially with reference to the 273 nm band, was obtained using the M06L functional (absolute value of Δ = 17.37 nm), B3LYP functional (absolute value of Δ = 20.18 nm), and the B3LYP/6311++G(d,p) approximation (absolute value of Δ = 20.94 nm). Whereas in the case of bifonazole **2** (Figure 7), with a reference to the experimental band 254 nm, the highest agreement was possible using the M06L, M062X, PBE1PBE, and wB97XD functionals (absolute value of Δ = 33.60 nm). It was also noted that in the case of bifonazole **2** for all functionals, the first band of absorption also corresponded to the maximum absorption in the theoretical UV spectrum (except for the use of the M06L functional, where the maximum absorption corresponded to the second band of absorption). It can also be concluded that the implementation of the wB97XD functional for **2** resulted in the formation of two explicit absorption bands. Therefore, the use of the functional wB97XD seems to be a favorable approach for the correct prediction of UV-vis spectra of the investigated bifonazole. The results of calculations involving the first excited states of **1** and **2** and using different functionals are collected in Table 5 and Table 6, Figure 6 and Figure 7, as well as in Tables S1 and S2 (Supplementary Material).

**Figure 8:**
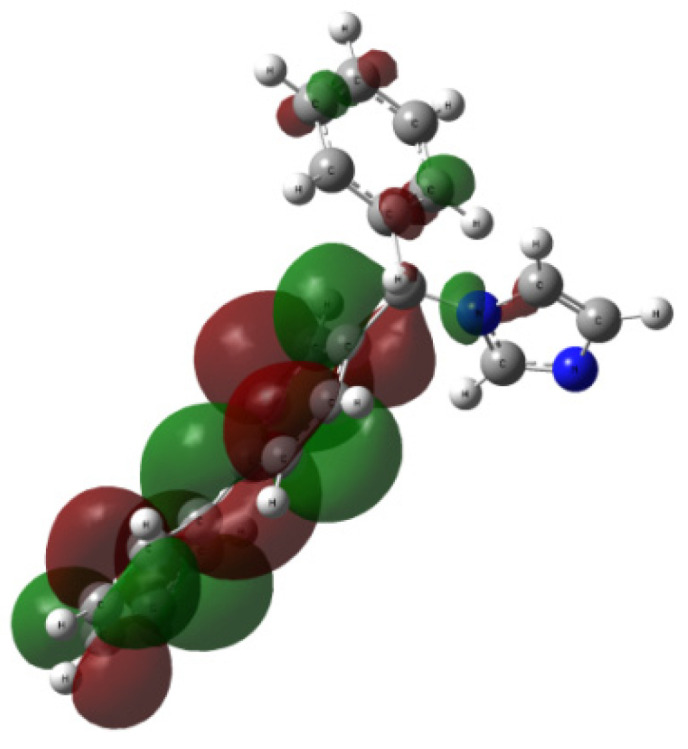
The HOMO orbitals generated for compound **1** (**up**; rotamer optimized at the B3LYP/6-31G(d,p) level of theory in methanol) and **2** (**down**; rotamer optimized at the wB97XD/6-31G(d,p) level of theory in methanol); vertical excited states calculated at the functional/6-311++G(2d,3p) level of theory (functional: B3LYP or wB97XD for **1** or **2**, respectively).

**Figure 9:**
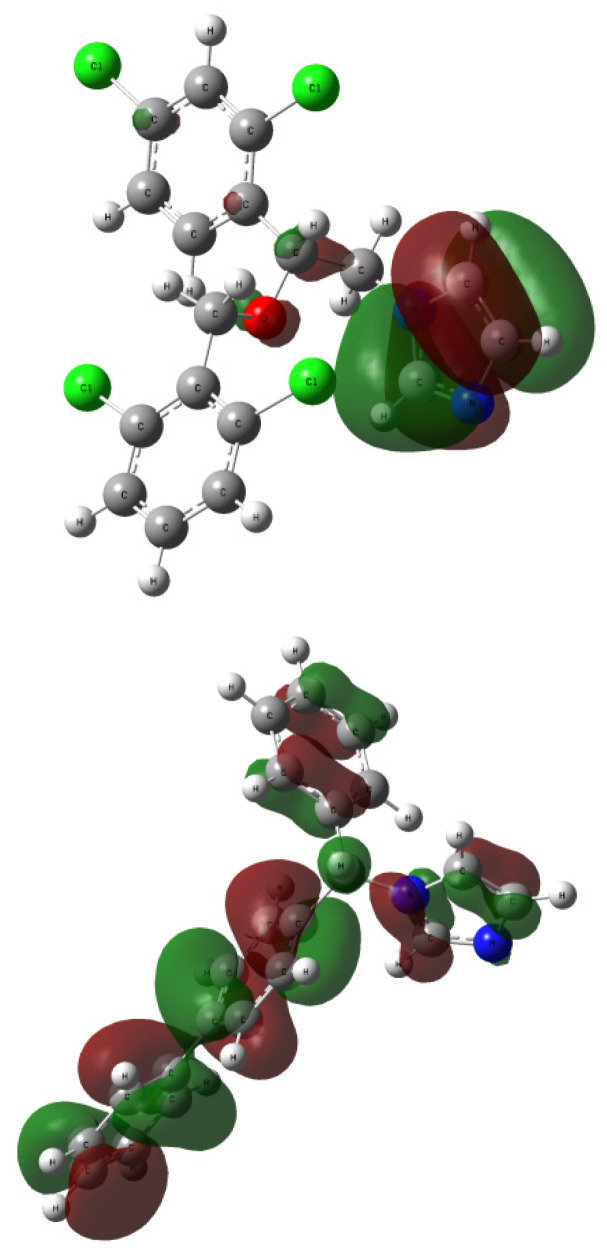
The LUMO orbitals generated for compound **1** (**up**; rotamer optimized at the B3LYP/6-31G(d,p) level of theory in methanol) and **2** (**down**; rotamer optimized at the wB97XD/6-31G(d,p) level of theory in methanol); vertical excited states calculated at the functional/6-311++G(2d,3p) level of theory (functional: B3LYP or wB97XD for **1** or **2**, respectively).

The contours of LUMO and HOMO orbitals for **1** and **2** (visualized based on the checkpoint file (.chk) generated during the TD-DFT computations) are presented in Figure 8 and Figure 9, respectively. The highest occupied molecular orbital (HOMO) is located mainly over all structure of analytes **1** and **2**, except for the azole moieties. The lowest unoccupied molecular orbital (LUMO) covers only the azole ring of **1** or all structure of the bifonazole **2**. It turned out that the isoconazole and bifonazole HOMO orbitals are not similar to the HOMO orbitals of posaconazole, itraconazole, voriconazole, and fluconazole. Dissimilarity between them is also related with the lowest occupied molecular orbital (LUMO) of **1**, which covers the only the diazole residue without dihalogenophenyl ring [6,7,23].

The HOMO–LUMO gap calculated for isoconazole **1** at the B3LYP/6-311++G(2d,3p) level is 5.2004 eV, corresponding to an electron transition from spinorbital 106 to spinorbital 107. It can be assigned to the calculated first excitation state at 263.64 nm (the HOMO−LUMO contribution relatively to the first excited state, calculated as duplicated coefficient square, is 99%, oscillator strength f = 0.0102, coefficient 0.70499, calculated energy is 4.4164 eV; data taken from the output file) and is slightly higher than for bifonazole **2** where that gap was estimated at 5.0181 eV [B3LYP/6-311++G(2d,3p)//B3LYP/6-31G(d,p) approach]. On the other hand, the HOMO–LUMO gap calculated for **2** at the wB97XD/6-311++G(2d,3p) level is 8.8147 eV is related to an electron transition from spinorbital 82 to spinorbital 83 and the first excitation state at 244.40 nm and is lower than for isoconazole **1** where that gap was estimated at 9.1657 eV (wB97XD/6-311++G(2d,3p)//wB97XD/6-31G(d,p) approach).

The first excited state for compound **1** (B3LYP/6-311++G(2d,3p)//B3LYP/6-31G(d,p) approach) relates mainly to the 263.64 nm band (oscillator strength f = 0.0102, coefficient 0.70499; data taken from the output file). In this case, the HOMO−LUMO contribution relatively to the first excited state is 99%. The first excited state for compound **2** (B3LYP/6-311++G(2d,3p)//B3LYP/6-31G(d,p) approach) relates mainly to the 241.62 nm band (the HOMO−LUMO contribution is 25%, HUMO-3 → LUMO+3, oscillator strength f = 0.0089, coefficient 0.49639, calculated energy is 5.1313 eV; data taken from the output file). In the case of bifonazole **2** (wB97XD/6-311++G(2d,3p)//wB97XD/6-31G(d,p) approach) the first excited state relates to the 244.40 nm band (contribution 87%, oscillator strength f = 0.8089, coefficient 0.65864; data taken from the output file). For **2**, however, using the B3LYP/6-311++G(2d,3p)//B3LYP/6-31G(d,p) approach, the first excited state relates to the 273.93 nm band (contribution 95%, oscillator strength f = 0.5857, coefficient 0.68914; data taken from the output file). Based on the data obtained, it can be concluded that the largest HO-MO-LUMO contribution for both isoconazole **1** and bifonazole **2** was observed when using functional B3LYP.

As mentioned, in the theoretical UV-vis spectrum of **1** (B3LYP/6-311++G(2d,3p)//B3LYP/6-31G(d,p) approach), the highest oscillator strength (f = 0.0102) can be assigned to the first excitation state at 263.647 nm. On the other hand, in the case of **2** (wB97XD/6-311++G(2d,3p)//wB97XD/6-31G(d,p) approach), the highest oscillator strength (f = 0.1255) can be assigned to the first excitation state at 244.40 nm (f = 0.8089). The above discussion shows that the DFT method can satisfactorily explain the observations taken from the experimental UV-vis spectra of the analyzed conazoles.

Next, for **1** and **2**, we computed several descriptors related to HOMO–LUMO electron transition, i.e., electronegativity (χ), chemical hardness (η) and electronic potential using the orbital energy of the HOMO and the orbital energy of the LUMO based on the DFT formalism, as well as the chemical potential (μ) of the molecule using Koopman’s theorem [24]. They are characterized by equations: μ = −(I + A)/2 and η = (I − A)/2, and electronegativity χ = (I + A)/2, where I is the first ionization potential (I = −EHOMO), A—electron affinity (A = −ELUMO). Regarding the above-mentioned data, these descriptors are as follows [eV]: I = 6.5520 or 6.4859, A = 1.3516 or 1.4678, μ = −3.9518 or −3.9768, η = 2.6002 or 2.5090, χ = 3.9518 or 3.9768 [B3LYP/6-311++G(2d,3p)//B3LYP/6-31G(d,p) approach], I = 6.5770 or 6.5011, A = 1.3489 or 1.4373, μ = −3.9630 or −3.9692, η = 2.6141 or 2.5319, χ = 3.9630 or 3.9692 [B3LYP/6-311++G(2d,3p)//B3LYP/6-311++G(d,p) approach], I = 8.5123 or 8.6038, A = 0.7418 or −0.1891, μ = −4.6271 or −4.2073, η = 3.8853 or 4.3964, χ = 4.6271 or 4.2073 [CAM-B3LYP/6-311++G(2d,3p)//CAM-B3LYP/6-31G(d,p) approach], I = 6.6960 or 7.8454, A = 1.2422 or 0.2414, μ = −3.9691 or −4.0434, η = 2.7269 or 3.8020, χ = 3.9691 or 4.0434 [APF/6-311++G(2d,3p)//APF/6-31G(d,p) approach], I = 6.7670 or 6.7194, A = 1.1530 or 1.3176, μ = −3.9600 or −4.0185, η = 2.8070 or 2.7009, χ = 3.9600 or 4.0185 [PBE1PBE/6-311++G(2d,3p)//PBE1PBE/6-31G(d,p) approach], I = 5.8557 or 5.8597, A = 1.7834 or 1.9614, μ = −3.8196 or −3.9106, η = 2.0361 or 1.9492, χ = 3.8196 or 3.9106 [M06L/6-311++G(2d,3p)//M06L/6-31G(d,p) approach], I = 7.8701 or 7.7874, A = 0.3739 or 0.6082, μ = −4.1220 or −4.1978, η = 3.7481 or 3.5896, χ = 4.1220 or 4.1978 [M062X/6-311++G(2d,3p)//M062X/6-31G(d,p) approach] and I = 8.5074 or 8.4288, A = −0.6582 or −0.3859, μ = −3.9246 or −4.0215, η = 4.5828 or 4.4073, χ = 3.9246 or 4.0215 [wB97XD/6-311++G(2d,3p)//wB97XD/6-31G(d,p) approach] for **1** or **2**, respectively. In case of **1** first ionization potential (I) reached higher values in comparison with **2** (except for the use of CAM-B3LYP, APF and functionals). Moreover, the use of wB97XD functional resulted in the largest value of this descriptor with respect to both azoles, as well as the fact that chemical potential (μ) had negative values (for the other functionals, μ values were positive).

With regard to other derivatives of **1** and **2** containing azole moiety with antifungial activity, i.e., voriconazole and fluconazole, and the above descriptors, we used in previous studies B3LYP/6-311++G(2d,3p)//B3LYP/6-31G(d,p) approach [23] or additionally computed at the wB97XD/6-311++G(2d,3p)//wB97XD/6-31G(d,p) level of theory. Regarding these approximations, these descriptors are as follows [eV]: I = 7.0718 or 7.1686, A = 2.0738 or 1.0806, μ = −4.5728 or −4.1246, η = 2.4990 or 3.0440, χ = 4.5728 or 4.1246 [B3LYP/6-311++G(2d,3p)//B3LYP/6-31G(d,p) approach], I = 9.0228 or 9.1069, A = 0.0974 or −0.8618, μ = −4.5601 or −4.1226, η = 4.4627 or 4.9843, χ = 4.5601 or 4.1226 [wB97XD/6-311++G(2d,3p)//wB97XD/6-31G(d,p) approach] for voriconazole or fluconazole, respectively. It was shown that the use of the functionalization of B3LYP or wB97XD results in smaller absolute values of the I and μ parameters compared to voriconazole or fluconazole in reference to **1** or **2**. In addition, the electron affinity parameter (A) computed for voriconazole achieved a higher value compared to the values estimated for **1** or **2**.

**Figure 10:**
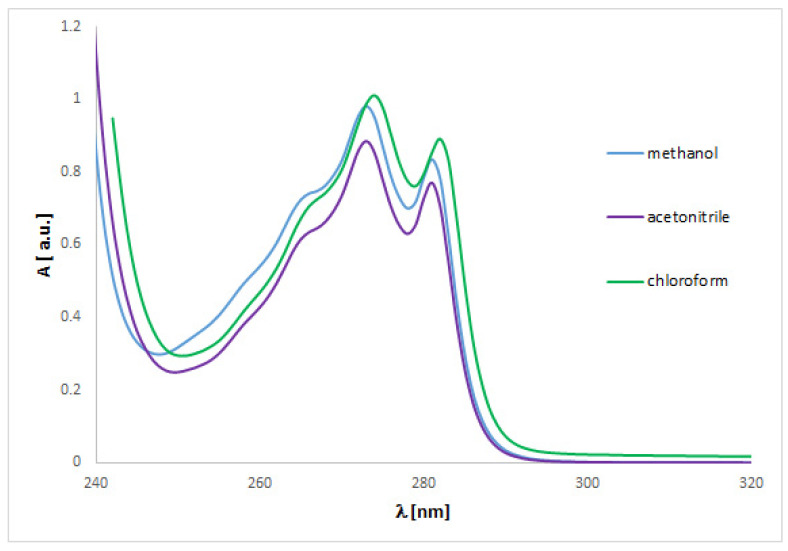
Experimental UV-vis spectrum of isoconazole **1** registered in methanol, acetonitrile and chloroform at 0.5 mg/mL.

**Figure 11:**
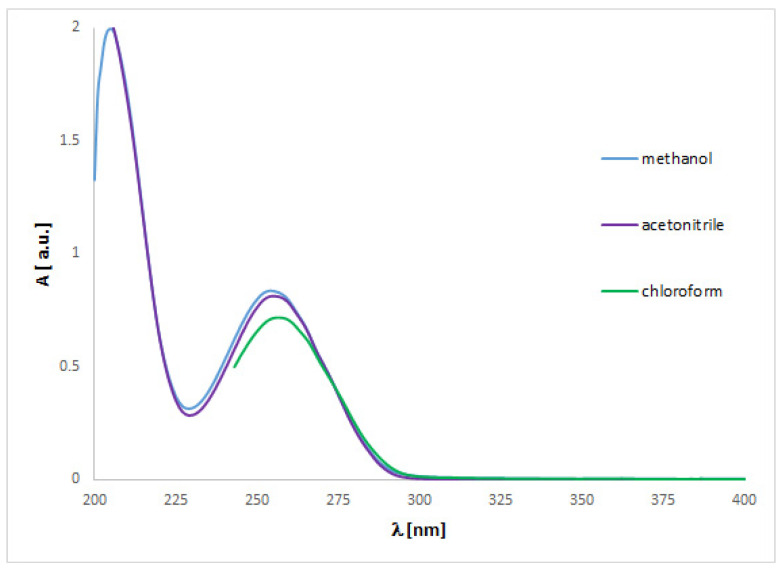
Experimental UV-vis spectrum of bifonazole **2** registered in methanol, acetonitrile and chloroform at 0.0025 mg/mL.

On the basis of the solvatochromism phenomenon, we decided to plot experimental UV-vis spectra for **1** and **2** including, in addition to methanol, the following solvents: chloroform and acetonitrile (Figure 10 and Figure 11). In the case of isoconazole **1**, the absorption band migrated at ca. 276 nm and was more shifted towards longer wavelengths in the case of chloroform (reaching the highest intensity), while it did not change in the methanol and acetonitrile medium. A similar phenomenon was observed for bifonazole **2** at ca. 254 nm, with the absorption band reaching a relatively lower intensity in the chloroform environment. Subsequently, using the TF-DFT method, theoretical UV-vis spectra were determined for **1** and **2** (Table 7 and Table 8). Analysis of the first excited state (λ_1_) and the λ_max_ bands of the theoretical UV-vis spectra of **1** (Table 7) or **2** (Table 8) indicates that the highest absorption values were observed when using M06L functional, and the lowest absorption values were observed for CAM-B3LYP functional. Besides, for **2**, the first excited state (λ_1_) and the λ_max_ bands reach the same values for the functional: CAM-B3LYP, PBE1PBE, M062X, and APF (in methanol and acetonitrile medium).

The discussion presented above provides important data relating to, e.g., the effect of reaction field and solvent polarity on the values of λ_max_ and first excited state (λ_1_) of theoretical UV-vis spectra of the azoles studied, depending on the functionals used for calculations. To the best of our knowledge, regarding this phenomenon, a wealth of experimental and theoretical approaches has not yet been studied in terms of azoles **1** and **2**.

### 2.4. NBO Analysis

Considering the conclusions drawn from the UV-vis analysis, we carried out natural bond orbitals (NBO) studies (CPCM solvation model and methanol used as solvent). The NBO analysis was performed at the wB97XD/6-311++G(2d,3p) level of theory using the NBO 3.0 approach as implemented in Gaussian G16 A.03 software for rotamers previously optimized in the wB97XD/6-31G(d,p) approximation. Our attention was focused on the oxygen and nitrogen atoms, as well as aromatic rings whose electrons were important for the distribution of HOMO and LUMO orbitals (Figure 1). The second order perturbation theory, which involves Fock matrix in the NBO basis, shows intramolecular hyper-conjugative interactions.

The fundamental structural differences between isoconazole **1** and bifonazole **2** are due to the presence of the two 1,3-chlororophenyl systems and oxygen atom bridge in the structure **1** compared to the other azole tested.

The C4-N2 bond in isoconazole (Figure 1) can be depicted by an almost completely filled (1.98666*e*) 2-centre bonding hybrid BD orbital (polarization coefficient 0.7961) formed by interaction between *s* (33.70% *s*) and *p* (66.27% *p*1.97) orbitals. The nitrogen atom has a greater contribution (63.37%) to this σ_C-N_ bonding orbital. The above bond in this rotamer is an NBO density donor to the following bonds formed by the antibonding orbitals BD*: N1-C1, N2-C1, N2-C2, C2-C3, and C5-C6. The C4-N2 bond also interacts with the antibonding Rydberg orbitals RY* of atoms: C1, C2, and C5. In comparison, the NBO characteristics concerning the analogous C-N bond in posaconazole, itraconazole voriconazole, and fluconazole is similar [6,7,23].

The O1-C12 bond in compound **1** (Figure 1) can be characterized by an almost completely filled (1.98838*e*) 2-centre hybrid bonding orbital (polarization coefficient 0.8222) formed by the overlap of *s* (28.27% *s*) and *p* (71.66% *p*2.53) orbitals. The oxygen atom has a greater contribution (67.60%) to the formation of this σ_O-N_ bonding orbital. This bond is also an NBO density donor to the following bonds formed by the antibonding orbital BD*: C4-C5, and C13-C14, as well as antibonding Rydberg orbitals RY* centered on atoms: C5, and C13. The analogous O-C bond in posaconazole, itraconazole voriconazole, and fluconazole [6,7,23] can be characterized similarly.

The N2-C1 bond within the azole ring in compound **1** (Figure 1) can be characterized by two almost completely filled (1.98607*e*) 2-centre bonding hybrid BD orbitals (polarization coefficients 0.8030 and 0.5960) formed by the overlap of: *s* (33.56% *s*) and *p* (66.41% *p*1.98) orbitals (in this bond the nitrogen atom has a greater contribution (64.48%) to the formation of this bonding orbital). This bond is also an NBO density donor to the following bonds formed by the antibonding orbitals BD*: N1, C2, C4, N2-C2, N2-C4, and C2-H2.

The N1-C7 bond in **2** (Figure 1), analogous to the N2-C4 bond in isoconazole **1**, can be characterized by an almost completely filled (1.98200*e*) 2-centre hybrid BD (polarization coefficient 0.7943) formed by the overlap of *s* (33.81% *s*) and *p* (66.16% *p*1.96) orbitals. The nitrogen atom has a greater contribution (63.10%) to the formation of the σ_N-C_ bonding orbital. This bond is also an NBO density donor to the following bonds formed by the antibonding orbitals BD*: N1-C20, N1-C22, N2-C20, C1-C6, C7-C8, C8-C9, and C21-C22, as well as the antibonding Rydberg orbitals RY* of atoms: C1, C8, C20, and C22.

The N1-C20 bond in the bifonazole **2**, analogous to the N2-C1 bond in isoconazole **1**, can be characterized by an almost filled (1.98539*e*) 2-centre hybrid BD (polarization coefficient 0.8027) formed by the overlap of s (33.39% s) and *p* (66.58% *p*1.99) orbitals. The nitrogen atom has a greater contribution (64.44%) to the formation of the σ_N-C_ bonding orbital. This bond is also an NBO density donor to the following bonds formed by the antibonding orbitals BD*: N1-C7, N1-C22, and C22-H18, as well as the antibonding Rydberg orbitals RY* of atoms: N2, C7, and C22.

Considering the above data, we can conclude that the distribution of the NBOs for rotamers of isoconazole and bifonazole almost identically covers especially the azole nitrogen atoms. The sole difference, discussed above, is connected with the NBO donor–acceptor interaction, including the hyper-conjugate interaction energy (Figure 1). The differences come down to the fact that a dichlorophenyl ring and -CH_2_-O-CH- bridge are present in the isoconazole structure as opposed to the bifonazole structure.

### 2.5. NMR Analysis

The signals in the ^1^H NMR spectra of isoconazole **1** and bifonazole **2** were registered in DMSO-*d6* (Table 9 and Table 10 and Figures S5a and S6d).

The theoretical ^1^H NMR spectra of **1** and **2** using the B3LYP functional (MAE = 0.25 or 0.24 for **1** or **2**, respectively; DMSO as solvent) show the highest conformity of the chemical shifts with the experimental data (Table 9). In the case of **1**, the largest values of percentage error (Δδ) more than 12% were found for the H5 and H10 methylene protons. These errors are due to steric reasons, namely proximity of the oxygen atom (closest distance CH_2_^…^O1 is 2.58 or 2.08 Å for the H5 or H10 atoms, respectively) or H1 atoms within the azole ring (distance CH_2_^…^H1 is 2.56 Å) and dichlorophenyl ring (distance CH_2_^…^Cl3 is 2.65 Å). Fundamentally, it should be emphasized that in the case of isoconazole **1**, the calculated values of chemical shifts in the ^1^H NMR spectrum presented significant correspondence with experimental data (MAE error range 0.25–0.64).

For bifonazole **2**, the compliance of the estimated values of chemical shifts with the experimental data of ^1^H NMR spectrum was expressed in the following ranges of MAE error values: 0.25−0.78. In the case of **2**, the theoretical ^1^H NMR spectra using the B3LYP functional (MAE = 0.25 for DMSO as solvent) show the highest conformity of the chemical shifts with the experimental data (Table 10). The highest values of percentage error (Δδ) exceeding ca. 8% were related to the H9 proton of the phenyl ring, which was caused by steric reasons. We noticed the proximity of the second phenyl rings (closest distance H9^...^H11 is ca. 2.33 Å). Moreover, the closest distance H6^...^H18 (within the azole ring) equaled ca. 2.58 Å and resulted in 6% percentage error (Δδ). The closest distance H6^...^H10 (within the phenyl ring) equaled ca. 2.38 Å and presented the error 6% too. The percentage error (Δδ) 7% was found for the H16 within the azole ring is due to proximity of the rotating phenyl ring (closest distance H16^…^C8 is 2.72 Å).

In consideration of the data presented, it should be emphasized that these data show that the DFT formalism, particularly the B3LYP functional, results in a correct description of the isoconazole and bifonazole ^1^H NMR chemical shifts, with the worst results being obtained using the M062X functional.

## 3. Materials and Methods

### 3.1. Chemicals

Isoconazole(**1**,ISO): (RS)-1-[2-[(2,6-Dichlorobenzyl)oxy]-2-(2,4-dichlorophenyl)ethyl]-1*H*-imidazole, was purchased from Shouguang Fukang Pharmaceutical Co., Ltd., Shouguang, China, purity ≥ 99% (in compliance with European Pharmacopoeia 8.0]).

Bifonazole (**2**, BIF): 1-([1,1′-Biphenyl]-4-ylphenylmethyl)-1*H*-imidazole, was purchased from Shouguang Fukang Pharmaceutical Co., Ltd., Shouguang, China, purity ≥ 99% (in compliance with European Pharmacopoeia 8.0).

### 3.2. Spectroscopy

The IR spectra were recorded in KBr (1.00 mg of compound 1 or 2 per 300 mg of KBr) on a Shimadzu IRAffinity-1 spectrometer.

The UV spectra were run on a Perkin Elmer UV/VIS Lambda 20 spectrophotometer in 1 cm quartz cuvettes using 0.15625, 0.03125, 0.0625, 0.125, 0.25 and 0.5 mg/mL solutions of compound 1 or 0.0003125, 0.000625, 0.00125, 0.0025, 0.005, 0.01 mg/mL solutions of compound 2 in methanol (all concentrations), chloroform (0.5 or 0.01 mg/mL for **1** or **2**, respectively), dichloromethane (0.5 or 0.01 mg/mL for 1 or 2, respectively), and acetonitrile (0.5 or 0.01 mg/mL for **1** or **2**, respectively).

The NMR spectra were recorded at 298 K on a NMR 700 MHz (16.44 T) AVANCE III Bruker spectrometer operating at 500 or NMR 500 MHz (11.74 T) AVANCE III Bruker spectrometer operating at 700 MHz (^1^H) and 126 MHz (^13^C). Bifonazole (10 mg) or isoconazole (10 mg) were dissolved in 500 μL of d_6_-DMSO (Aldrich). TMS was used as an internal standard.

Isoconazole (**1**): ^1^H NMR (500 MHz, DMSO-*d6*) δ 7.68 (d, J = 1.8 Hz, 1H), 7.49 (dd, J = 8.4, 1.9 Hz, 1H), 7.46 (d, J = 3.1 Hz, 2H), 7.45 (s, 1H), 7.44 (d, J = 3.5, 1H), 7.38 (dd, J = 8.8, 7.2 Hz, 1H), 7.01 (s, 1H), 6.82 (s, 1H), 5.06 (dd, J = 6.8, 4.1 Hz, 1H), 4.62 (d, J = 10.8 Hz, 1H), 4.56 (d, J = 10.8 Hz, 1H), 4.28–4.14 (m, 2H).

Bifanozole (**2**): ^1^H NMR (700 MHz, DMSO-*d6*) δ 7.70–7.68 (d, J = 8.3 Hz, 2H), 7.69 (s, 1H), 7.67 (d, J = 7.3 Hz, 2H), 7.47 (t, J = 7.7 Hz, 2H), 7.41 (t, J = 7.5 Hz, 2H), 7.38–7.35 (q, J = 7.4, 2H), 7.21 (dd, J = 18.8, 7.8 Hz, 4H), 7.15 (s, 1H), 6.98 (s, 1H), 6.93 (s, 1H).

High resolution mass spectrometry analysis was performed using Q-Exactive Orbitrap mass spectrometer (Thermo Fisher Scientific, Bremen, Germany) equipped with TriVersa NanoMate ESI ion source (Advion BioSciences ltd., Ithaca, NY, USA) working in direct infusion mode. 5 µL sample aliquots were infused directly into mass spectrometer, after ion current stabilization, spectra were acquired for 5 min. TriVersa source was operating at 1.25 psi nitrogen pressure and ionization voltage was set to 1.05 kV. HRMS data were collected in positive ion mode within the range of m/z 100–1500 at the resolution of 140,000 (at m/z 200, full width at half maximum, FWHM). All analyses were performed using automatic gain control (AGC) set to target value of 3 × 106 and ion injection time (IT) was set to 100 ms.

The ^1^H, ^13^C, H-H COSY, H-C HSQC, and HR MS spectra of **1** or **2** are given in the supplementary material (Figures S5a–S8).

### 3.3. Theoretical Calculations

The initial structures of optimized rotamers of isoconazole 1 and bifonazole 2 were taken from the *.cif files given in the Cambridge Structural Database (CSD) hosted by the Cambridge Crystallographic Data Centre (CCDC) for 1 [25] and 2 [26]. They were initially optimized (Gaussian 16 A.03 program [27]) using DFT formalism [28], namely: (a) B3LYP/6-31G(d,p) [29], (b) CAM-B3LYP/6-31G(d,p) [30], (c) B3LYP/6-311+G(d,p) [31,32], (d) PBE1PBE/6-31G(d,p) [33] (e) M06L/6-31G(d,p) [34], M062X/6-31G(d,p) [35] (f) wB97XD/6-31G(d,p) [36], and (g) APF/6-31G(d,p) approaches in the gaseous phase (IR and UV spectrum calculations) or by applying the CPCM model [37,38] (UV and NMR spectrum calculations). For UV-vis calculations, we applied the TD-DFT method, CPCM solvation model, the linear response (LR) approach, and solvents: chloroform, methanol, and acetonitrile. The NMR shift for the TMS reference proton (Href) was calculated by the a−g approaches in DMSO at 293 K using the gauge-including atomic orbital (GIAO) method [39] implemented in Gaussian G16 A.03 program and the protocol described in our previous report [6,7,8,23]. The Chemcraft 1.7 software was utilized for the visualization of all optimized rotamers [40]. The HOMO–LUMO orbitals for compounds were generated based on checkpoint files using GaussView 5.0 program [41]. For the theoretical IR and UV-vis spectra visualization, we used the Gabedit 2.3.4 software [42] with default settings (‘Lorentzian lineshape’ option). Calculations were carried out using resources provided by Wrocław Center for Networking and Supercomputing.

## 4. Conclusions

Our computations proved that the use of the CAM-B3LYP or B3LYP functional seems to be comparatively more effective in the IR spectra predictions of isoconazole **1** and bifonazole **2** because these approaches generally afford results without significant errors. The best conformity with the experimental UV spectra was obtained with the use of B3LYP/6-31G(d,p) (for isoconazole **1**) or wB97XD/6-31G(d,p (for bifonazole **2**) methods. The HOMO–LUMO gap calculated for isoconazole **1** at the B3LYP/6-311++G(2d,3p) level is 5.2004 eV, corresponding to an electron transition from spinorbital 106 to spinorbital 107. It can be assigned to the calculated first excitation state at 263.64 nm (the HOMO−LUMO contribution relative to the first excited state, calculated as duplicated coefficient square, is 99%, oscillator strength f = 0.0102, coefficient 0.70499, calculated energy is 4.4164 eV; data taken from the output file) and is slightly higher than for bifonazole **2**, where that gap was estimated at 5.0181 eV (B3LYP/6-311++G(2d,3p)//B3LYP/6-31G(d,p) approach). On the other hand, the HOMO–LUMO gap calculated for **2** at the wB97XD/6-311++G(2d,3p) level is 8.8147 eV is related to an electron transition from spinorbital 82 to spinorbital 83 and the first excitation state at 244.40 nm and is lower than for isoconazole **1** where that gap was estimated at 9.1657 eV (wB97XD/6-311++G(2d,3p)//wB97XD/6-31G(d,p) approach). For **1** and **2**, we computed several descriptors related to HOMO–LUMO electron transition, i.e., electronegativity (χ), chemical hardness (η), and electronic potential, using the orbital energy of the HOMO and the orbital energy of the LUMO based on the DFT formalism, as well as the chemical potential (μ) of the molecule using Koopman’s theorem. For **1**, first ionization potential (I) reached higher values compared to **2** (except for the use of CAM-B3LYP, APF and functionals). In addition, the use of wB97XD functional resulted in the largest value of this descriptor relative to both azoles, as well as the fact that chemical potential (μ) took negative values (for the other functionals, μ values were positive). Moreover, analysis of the first excited state (λ_1_) and the λ_max_ bands of the theoretical UV-vis spectra of **1** or **2** indicates that the highest absorption values were observed for the use of the M06L functional, and the lowest for the CAM-B3LYP functional. Besides, for **2**, the first excited state (λ_1_) and the λ_max_ bands reach the same values for the functional: CAM-B3LYP, PBE1PBE, M062X and APF (in methanol and acetonitrile medium). In our work we compared ^1^H NMR experimental and theoretical spectra of **1** and **2**. The calculated data show that the DFT formalism, particularly B3LYP functionals, result in a correct description of the isoconazole and bifonazole ^1^H NMR chemical shifts. In the case of **1** the largest values of percentage error (Δδ) to be more than 12% were found for the H5 and H10 methylene protons. These errors are due to steric reasons, namely proximity of the oxygen atom (closest distance CH_2_^…^O1 is 2.58 or 2.08 Å for the H5 or H10 atoms, respectively) and H1 atoms within the azole ring (distance CH_2_^…^H1 is 2.56 Å) and dichlorophenyl ring (distance CH_2_^…^Cl3 is 2.65 Å). In the case of isoconazole **1**, the calculated values of chemical shifts in the ^1^H NMR spectrum gave significant correspondence with experimental data (MAE error ranged from 0.25−0.64). For bifonazole **2**, the compliance of the estimated values of chemical shifts with the experimental data of the ^1^H NMR spectrum was expressed in the following ranges of MAE error values: 0.25−0.78. In the case of **2**, the theoretical ^1^H NMR spectra using the B3LYP functional (MAE = 0.25 for DMSO as solvent) show the highest conformity of the chemical shifts with the experimental data. The highest values of percentage error (Δδ) exceeding ca. 8% were related to the H9 proton of the phenyl ring, which was caused by steric reasons. The above conclusions show that our proposed methodology seems to be a potentially useful tool for the prediction of IR and UV-vis properties of biologically active conazoles. We wish to investigate this standpoint further in the near future, additionally involving the tuned range-separated functionals [43,44,45,46,47] (especially regarding the TD-DFT computations and all investigated so far azoles [6,7,8,23]).

## Figures and Tables

**Table 1:** The atomic charges [*e*] for nitrogen, chloride and oxygen atoms within the structure of isconazole (geometry optimized at the B3LYP/6-31G(d,p) or CAM-B3LYP/6-31G(d,p) levels of theory in gaseous phase, the Mulliken, CHelpG and NBO charges computations at the B3LYP/6-311++G(2d,3p) or CAM-B3LYP/6-311++G(2d,3p) levels of theory) estimated according to the Mulliken, CHelpG or NBO methodology (atoms numbering as in the Figure 1).

Heteroatom	Calculated Charges					
	Mulliken		CHelpG		NBO	
	B3LYP	CAM-B3LYP	B3LYP	CAM-B3LYP	B3LYP	CAM-B3LYP
**N1**	−0.317	−0.289	−0.572	−0.607	−0.503	−0.510
**N2**	0.080	0.111	0.286	0.178	−0.421	−0.421
**O1**	−0.362	−0.376	−0.395	−0.406	−0.604	−0.610
**Cl1**	−0.016	−0.044	−0.112	−0.112	0.012	0.012
**Cl2**	0.107	0.066	−0.123	−0.127	0.022	0.021
**Cl3**	0.001	−0.005	−0.073	−0.079	0.034	0.037
**Cl4**	0.136	0.072	−0.105	−0.104	0.011	0.008

**Table 2:** The atomic charges [*e*] for nitrogen atoms within the structure of bifonazole (geometry optimized at the B3LYP/6-31G(d,p) or CAM-B3LYP/6-31G(d,p) levels of theory in gaseous phase, the Mulliken, CHelpG and NBO charges computations at the B3LYP/6-311++G(2d,3p) or CAM-B3LYP/6-311++G(2d,3p) levels of theory) estimated according to the Mulliken, CHelpG or NBO methodology (atoms numbering as in the Figure 1).

Heteroatom	Calculated Charges					
	Mulliken		CHelpG		NBO	
	B3LYP	CAM-B3LYP	B3LYP	CAM-B3LYP	B3LYP	CAM-B3LYP
**N1**	0.050	0.033	0.173	0.169	−0.447	−0.448
**N2**	−0.345	−0.334	−0.588	−0.596	−0.502	−0.507

**Table 3:** IR spectrum of compound **1**; CAM-B3LYP/6-31G(d,p)/gas level of theory; υ: stretching; δ: in-plane bending; γ: out-of-plane bending.

IR Spectrum of Isoconazole 1		
Experimental Wavenumber (cm^−1^)	Vibrational Assignments	Calculated Wavenumber (cm^−1^)
		CAM-B3LYP/6-31G(d,p)
3121, 3119, 3117, 3115, 3113	ν C-H arom	3301, 3282, 3273, 3257, 3254, 3250, 3249, 3234, 3227
2939, 2895	ν C-H alkyl	3166, 3124, 3107, 3074, 3052
1683, 1647	ν C-C arom	1686, 1682, 1658, 1653
1581, 1560, 1435	ν N=N and ν C-N azole	1593, 1573
1348	δ C-N azole	1342, 1336
1279, 1230	δ C-H arom	1288, 1278, 1263, 1260, 1244, 1232, 1200
1105	ν C-O-C asym	1192
1072, 1043, 1016	ν C-O-C sym (alkyl-aryl ether and cyclic ether) and ν C-O-C asym (cyclic ether)	1080, 1074
905, 781, 764, 743, 660	γ C-H benzene and azole	931, 930, 905, 897, 883, 870, 867, 839, 809, 746, 682
868, 827, 806	ν C-Cl arom	897, 815, 682

**Table 4:** IR spectrum of compound **2**; B3LYP/6-31G(d,p)/gas level of theory; υ: stretching; δ: in-plane bending; γ: out-of-plane bending.

IR Spectrum of Bifonazole 2		
Experimental Wavenumber (cm^−1^)	Vibrational Assignments	Calculated Wavenumber (cm^−1^)
		B3LYP/6-31G(d,p)
3122, 3082, 3032	ν C-H arom	3281, 3275, 3248, 3210, 3208, 3203, 3201, 3198, 3193, 3192, 3190, 3183, 3182, 3177, 3171
2929	ν C-H alkyl	3043
1491	ν C-C arom	1494
1641, 1614	ν N=N and ν C-N triazole	1530
1221	δ C-H arom	1380, 1368, 1350, 1343, 1318, 1315, 1312, 1238, 1221, 1218
1348	δ C-N azole	1396
760, 725	γ C-H benzene and azole	776, 772, 745, 730, 726714, 676

**Table 5:** First excited states of the isoconazole **1** computed using LR TD DFT approach in vacuum or in methanol; B3LYP/6-311++G(2d,3p)//B3LYP/6-31G(d,p) level of theory.

Compound 1				
Environment	Energy [eV]	Wavelength [nm]	Oscillator Strength	Ground State−First Excited State Orbital Transition
Vacuum	5.0922	243.48	0.0086	106 → 107
Methanol	4.7028	263.64	0.0102	

**Table 6:** First excited states of the bifonazole **2** computed using LR TD DFT approach in vacuum or in methanol; wB97XD/6-311++G(2d,3p)//wB97XD/6-31G(d,p) level of theory.

Compound 2				
Environment	Energy [eV]	Wavelength [nm]	Oscillator Strength	Ground State−First Excited State Orbital Transition
Vacuum	5.1333	241.53	0.1976	82 → 83
Methanol	5.0730	244.40	0.8089	

**Table 7:** Calculated wavelength for first excited state (λ_1_) and maximum of absorption (λ_max_) for isoconazole **1** using different functionals: **B3LYP**—B3LYP/6-31G(d,p); **6311G(d,p)**—B3LYP/6-311++G(d,p); **CAM**—CAM-B3LYP/6-31G(d,p); **APF**—APF/6-31G(d,p); **M06L**—M06L/6-31G(d,p); **M062X**—M062X/6-31G(d,p), **PBE1PBE**—PBE1PBE/6-31G(d,p); **wB97XD**—wB97XD/6-31G(d,p). For UV-vis calculations were used TD-DFT method, CPCM solvation model, the linear response (LR) approach and solvents: methanol, acetonitrile and chloroform.

**λ_1_**								
**Solvent/Functional**	**CAM**	**PBE1PBE**	**M06L**	**B3LYP**	**APF**	**6311G(d,p)**	**M062X**	**wB97XD**
methanol	240.58	248.82	304.26	263.64	254.14	261.88	238.95	241.62
acetonitrile	240.59	248.76	304.17	263.56	254.07	261.81	238.96	241.62
chloroform	241.01	253.14	311.45	268.73	258.74	266.99	239.34	242.03
**λ_max_**								
**Solvent/Functional**	**CAM**	**PBE1PBE**	**M06L**	**B3LYP**	**APF**	**6311G(d,p)**	**M062X**	**wB97XD**
methanol	197.69	224.75	262.92	229.59	227.55	229.37	213.65	198.12
acetonitrile	197.77	224.78	262.00	229.61	227.58	229.37	213.68	198.21
chloroform	199.01	225.44	259.83	232.79	227.47	231.68	214.30	199.13

**Table 8:** Calculated wavelength for first excited state (λ_1_) and maximum of absorption (λ_max_) for bifonazole **1** using different functionals: **B3LYP**—B3LYP/6-31G(d,p); **6311G(d,p)**—B3LYP/6-311++G(d,p); **CAM**—CAM-B3LYP/6-31G(d,p); **APF**—APF/6-31G(d,p); **M06L**—M06L/6-31G(d,p); **M062X**—M062X/6-31G(d,p), **PBE1PBE**—PBE1PBE/6-31G(d,p); **wB97XD**—wB97XD/6-31G(d,p). For UV-vis calculations were used TD-DFT method, CPCM solvation model, the linear response (LR) approach and solvents: methanol, acetonitrile and chloroform.

**λ_1_**								
**Solvent/Functional**	**CAM**	**PBE1PBE**	**M06L**	**B3LYP**	**APF**	**6311G(d,p)**	**M062X**	**wB97XD**
methanol	248.61	264.90	313.92	273.93	267.17	271.58	247.18	244.40
acetonitrile	248.71	265.01	313.85	274.01	267.27	271.65	247.29	244.50
chloroform	249.12	265.79	318.82	276.00	268.58	273.69	247.72	244.92
**λ_max_**								
**Solvent/Functional**	**CAM**	**PBE1PBE**	**M06L**	**B3LYP**	**APF**	**6311G(d,p)**	**M062X**	**wB97XD**
methanol	248.61	264.90	287.60	272.53	267.17	267.17	247.18	244.40
acetonitrile	248.71	265.01	287.70	272.51	267.27	267.17	247.29	244.50
chloroform	249.12	265.79	288.29	273.13	264.48	269.08	247.72	244.92

**Table 9:** Experimental (δ_exp_) and calculated chemical shifts (**I**) for compound **1**; errors (Δ), relative percentage errors (Δδ); calculated NMR shielding (B3LYP/631G(d,p)//B3LYP/6-31G(d,p)/DMSO) for proton H_ref_ = 31.7468 ppm for TMS; MAD = 0.25 (atoms numbering as is in Figure 1).

Atoms Numbering	δexp	I	Δ	Δδ
H1	7.45	7.27	0.18	3
H2	7.01	7.30	−0.29	4
H3	6.82	7.13	−0.31	4
H4	4.21	3.93	0.28	7
H5	4.21	3.72	0.49	13
H6	5.06	4.93	0.13	3
H7	7.68	7.40	0.28	4
H8	7.49	7.49	0.00	0
H9	7.44	8.15	−0.71	9
H10	4.62	4.13	0.49	12
H11	4.56	4.57	−0.01	0
H12	7.46	7.35	0.11	1
H13	7.38	7.50	−0.12	2
H14	7.46	7.40	0.06	1

**Table 10:** Experimental (δ_exp_) and calculated chemical shifts (**I**) for compound **2**; errors (Δ), relative percentage errors (Δδ); calculated NMR shielding (B3LYP/631G(d,p)//B3LYP/6-31G(d,p)/DMSO) for proton H_ref_ = 31.7468 ppm for TMS; MAD = 0.24 (atoms numbering as is in Figure 1).

Atoms Numbering	δexp	I	Δ	Δδ
H1	7.21	6.97	0.24	3
H2	7.41	7.56	−0.15	2
H3	7.36	7.52	−0.16	2
H4	7.41	7.57	−0.16	2
H5	7.21	7.46	−0.25	3
H6	6.93	6.56	0.37	6
H7	7.69	7.25	0.44	6
H8	7.21	7.65	−0.44	6
H9	7.21	7.80	−0.59	8
H10	7.69	7.78	−0.09	1
H11	7.67	7.74	−0.07	1
H12	7.47	7.65	−0.18	2
H13	7.36	7.55	−0.19	2
H14	7.47	7.64	−0.17	2
H15	7.67	7.74	−0.07	1
H16	7.69	7.16	0.53	7
H17	6.98	7.17	−0.19	3
H18	7.15	7.15	−0.00	0

## Data Availability

Data is contained within the article and supplementary material.

## Supplementary Materials

The following are available online at https://www.mdpi.com/article/10.3390/ijms24010520/s1. The supplement contains Cartesian coordinates of the rotamers and complete results of the UV-vis, and NMR spectra and calculations.
