# Supplementary material for: Structural and Spectroscopic Properties of Isoconazole and Bifonazole—Experimental and Theoretical Studies
Source: Int J Mol Sci. 2022 Dec 28;24(1):520. doi: 10.3390/ijms24010520 (PMC9820235; doi:10.3390/ijms24010520)

Structural and Spectroscopic Properties of Isoconazole and Bifonazole—Experimental and Theoretical Studies

Beata Drabińska [1], Katarzyna Dettlaff [2], Tomasz Ratajczak [3,4,5], Kacper Kossakowski [1], Marcin K. Chmielewski [6], Judyta Cielecka-Piontek [7,*] and Jacek Kujawski [1,5,*]

[1] *Chair and Department of Organic Chemistry, Faculty of Pharmacy, Poznan University of Medical Sciences, Grunwaldzka 6 Str., 60-780 Poznań, Poland*

[2] *Chair and Department of Pharmaceutical Chemistry, Faculty of Pharmacy, Poznan University of Medical Sciences, Grunwaldzka 6 Str., 60-780 Poznań, Poland*

[3] *Liquid Dosage Form Laboratory, Research and Development Department, Polfa Warszawa S.A, Karolkowa 22/24 Str., 01-207, Warsaw, Poland*

[4] *Centre of New Technologies, University of Warsaw, Banacha 2C Str., 02-097 Warsaw, Poland*

[5] *Biosynthesis Sp. z o.o., Rubież 46 Str., 61-612 Poznań, Poland*

[6] *Institute of Bioorganic Chemistry, Polish Academy of Sciences, Noskowskiego 12/14 Str., 61-704 Poznań, Poland*

[7] *Chair and Department of Pharmacognosy, Faculty of Pharmacy, Poznan University of Medical Sciences, Rokietnicka 3 Str., 60-780 Poznań, Poland*

* *Correspondence: pionetk@ump.edu.pl (J.C.-P.); jacekkuj@ump.edu.pl (J.K.)*

IR spectra

UV-vis spectra were generated based on **.log* files obtained from calculations using the Gaussian package. For visualization we used the *Gabedit 2.3.4* software with default settings ('Lorentzian lineshape' instead of 'Gaussian lineshape' option with 'Set ymax=1' settings). *ASCII* files were generated using it, which were then opened with *Excel* software (MS Windows). The experimental spectra scaled at ≤ 1 were exported from the spectrophotometer software (*UV WinLab 2.70.01*, Perkin-Elmer Corporation) and overlaid along with the data (data from *ASCII* files) obtained from the calculations onto a single graph in Excel software (MS Windows).

Since the experimental spectra were presented in terms of transmittance (T) expressed as a percentage, rather than absorbance (A), the data obtained from the calculations (data from *ASCII* files taken from the corresponding **.log* files; *Gaussian* package) were recalculated using the relationship between these quantities:

$$A = \log\frac{100}{T}$$

$$T = (10^{-A}) \times 100\ [\%]$$

and together with the experimental spectra exported from the spectrophotometer software (*IR Solution 1.40*, Shimadzu Corporation) were overlaid on a single graph in Excel (MS Windows). To increase the readability of the graph, successive spectra were staggered by 70 units [%] of transmittance.

UV spectra

UV-vis spectra were generated based on **.log* files obtained from calculations using the *Gaussian* package. For visualization we used the *Gabedit 2.3.4* software with default settings ('Lorentzian lineshape' instead of 'Gaussian lineshape' option with 'Set ymax=1' settings). *ASCII* files were generated with this software, which were then opened with *Excel* software (MS Windows). Experimental spectra scaled at ≤ 1 were exported from the spectrophotometer software (*UV WinLab 2.70.01*, Perkin-Elmer Corporation) and overlaid with the data (data from *ASCII* files) obtained from the calculations onto a single graph in *Excel* software (MS Windows).

Table S1. Experimental (**δexp**) and theoretical (**I**) chemical shifts for compound **1**; errors (Δ). relative percentage errors (δ); calculated NMR shielding: (**B3LYP/6-311++G(d.p)/DMSO**) for proton H_{ref} = 32.0022 ppm for TMS. MAD = 0.27; (**CAM-B3LYP/6-31G(d.p)/DMSO**) for proton H_{ref} = 31.7664 ppm for TMS. MAD = 0.28; (**APF/6-31G(d.p)/DMSO**) for proton H_{ref} = 31.6674 ppm for TMS. MAD = 0.27; (**M06L/6-31G(d.p)/DMSO**) for proton H_{ref} = 31.9537 ppm for TMS. MAD = 0.36 (table in main manuscript); (**PBE1PBE/6-31G(d.p)/DMSO**) for proton H_{ref} = 31.6526 ppm for TMS. MAD = 0.27; **(M062X/6-31G(d,p)/DMSO)** for proton H_{ref} = 31.953 ppm for TMS. MAD = 0.64; **(wB97XD/6-31G(d,p)/DMSO)** for proton H_{ref} = 31.782 ppm for TMS. MAD = 0.25.

B3LYP/6-311++G(d,p)/DMSO				
**Atoms numbering**	**I**	**δexp**	**Δ**	**Δδ**
**H1**	7.49	7.45	-0.04	1%
**H2**	7.57	7.01	-0.56	7%
**H3**	7.39	6.82	-0.57	8%
**H4**	4.13	4.21	0.08	2%
**H5**	3.93	4.21	0.28	7%
**H6**	5.27	5.06	-0.21	4%
**H7**	7.62	7.68	0.06	1%
**H8**	7.7	7.49	-0.21	3%
**H9**	8.32	7.44	-0.88	11%
**H10**	4.39	4.62	0.23	5%
**H11**	4.75	4.56	-0.19	4%
**H12**	7.55	7.46	-0.09	1%
**H13**	7.71	7.38	-0.33	4%
**H14**	7.6	7.46	-0.14	2%

CAM-B3LYP/6-31G(d,p)/DMSO				
**Atoms numbering**	**I**	**δexp**	**Δ**	**Δδ**
**H1**	7.3	7.45	0.15	2%
**H2**	7.38	7.01	-0.37	5%
**H3**	7.18	6.82	-0.36	5%
**H4**	3.94	4.21	0.27	7%
**H5**	3.7	4.21	0.51	14%
**H6**	4.85	5.06	0.21	4%
**H7**	7.52	7.68	0.16	2%
**H8**	7.63	7.49	-0.14	2%
**H9**	8.33	7.44	-0.89	11%
**H10**	4.11	4.62	0.51	12%
**H11**	4.57	4.56	-0.01	0%
**H12**	7.49	7.46	-0.03	0%
**H13**	7.63	7.38	-0.25	3%
**H14**	7.54	7.46	-0.08	1%

APF/6-31G(d,p)/DMSO				
**Atoms numbering**	**I**	**δexp**	**Δ**	**Δδ**
**H1**	7.33	7.45	0.12	2%
**H2**	7.43	7.01	-0.42	6%
**H3**	7.23	6.82	-0.41	6%
**H4**	3.99	4.21	0.22	5%
**H5**	3.76	4.21	0.45	12%
**H6**	4.99	5.06	0.07	1%
**H7**	7.55	7.68	0.13	2%
**H8**	7.63	7.49	-0.14	2%
**H9**	8.3	7.44	-0.86	10%
**H10**	4.12	4.62	0.5	12%
**H11**	4.59	4.56	-0.03	1%
**H12**	7.49	7.46	-0.03	0%
**H13**	7.64	7.38	-0.26	3%
**H14**	7.53	7.46	-0.07	1%

M06L/6-31G(d,p)/DMSO				
**Atoms numbering**	**I**	**δexp**	**Δ**	**Δδ**
**H1**	7.29	7.45	0.16	2%
**H2**	7.62	7.01	-0.61	8%
**H3**	7.42	6.82	-0.6	8%
**H4**	4.31	4.21	-0.1	2%
**H5**	3.66	4.21	0.55	15%
**H6**	5.55	5.06	-0.49	9%
**H7**	6.99	7.68	0.69	10%
**H8**	7.72	7.49	-0.23	3%
**H9**	7.99	7.44	-0.55	7%
**H10**	4.03	4.62	0.59	15%
**H11**	4.81	4.56	-0.25	5%
**H12**	7.42	7.46	0.04	1%
**H13**	7.48	7.38	-0.1	1%
**H14**	7.42	7.46	0.04	1%

PBE1PBE/6-31G(d,p)/DMSO				
**Atoms numbering**	I	δexp	Δ	Δδ
**H1**	7.33	7.45	0.12	2%
**H2**	7.45	7.01	-0.44	6%
**H3**	7.25	6.82	-0.43	6%
**H4**	4.01	4.21	0.2	5%
**H5**	3.74	4.21	0.47	13%
**H6**	4.98	5.06	0.08	2%
**H7**	7.56	7.68	0.12	2%
**H8**	7.66	7.49	-0.17	2%
**H9**	8.32	7.44	-0.88	11%
**H10**	4.11	4.62	0.51	12%
**H11**	4.59	4.56	-0.03	1%
**H12**	7.48	7.46	-0.02	0%
**H13**	7.65	7.38	-0.27	4%
**H14**	7.55	7.46	-0.09	1%

M062X/6-31G(d,p)/DMSO				
**Atoms numbering**	I	δexp	Δ	Δδ
**H1**	7.66	7.45	-0.21	3%
**H2**	7.7	7.01	-0.69	9%
**H3**	7.86	6.82	-1.04	13%
**H4**	3.8	4.21	0.41	11%
**H5**	3.67	4.21	0.54	15%
**H6**	4.87	5.06	0.19	4%
**H7**	8.33	7.68	-0.65	8%
**H8**	8.34	7.49	-0.85	10%
**H9**	8.76	7.44	-1.32	15%
**H10**	4	4.62	0.62	15%
**H11**	5.17	4.56	-0.61	12%
**H12**	7.93	7.46	-0.47	6%
**H13**	8.23	7.38	-0.85	10%
**H14**	8.03	7.46	-0.57	7%

wB97XD/6-31G(d,p)/DMSO				
**Atoms numbering**	I	δexp	Δ	Δδ
**H1**	7.46	7.45	-0.01	0%
**H2**	6.75	7.01	0.26	4%
**H3**	6.51	6.82	0.31	5%
**H4**	3.94	4.21	0.27	7%
**H5**	3.63	4.21	0.58	16%
**H6**	4.83	5.06	0.23	5%
**H7**	7.4	7.68	0.28	4%
**H8**	7.71	7.49	-0.22	3%
**H9**	8.24	7.44	-0.8	10%
**H10**	4.44	4.62	0.18	4%
**H11**	4.52	4.56	0.04	1%
**H12**	7.49	7.46	-0.03	0%
**H13**	7.57	7.38	-0.19	3%
**H14**	7.4	7.46	0.06	1%

Table S2. Experimental (**δexp**) and theoretical (**I**) chemical shifts for compound **2**; errors (Δ). relative percentage errors (δ); calculated NMR shielding: (**B3LYP/6-311++G(d.p)/DMSO**) for proton H_{ref} = 32.0022 ppm for TMS. MAD = 0.35; (**CAM-B3LYP/6-31G(d.p)/DMSO**) for proton H_{ref} = 31.7664 ppm for TMS. MAD = 0.32; (**APF/6-31G(d.p)/DMSO**) for proton H_{ref} = 31.6674 ppm for TMS. MAD = 0.34; (**M06L/6-31G(d.p)/DMSO**) for proton H_{ref} = 31.9537 ppm for TMS. MAD = 0.3; (**PBE1PBE/6-31G(d.p)/DMSO**) for proton H_{ref} = 31.6526 ppm for TMS. MAD = 0.36; **(M062X/6-31G(d,p)/DMSO)** for proton H_{ref} = 31.953 ppm for TMS. MAD = 0.78; **(wB97XD/6-31G(d,p)/DMSO)** for proton H_{ref} = 31.782 ppm for TMS. MAD = 0.38.

B3LYP/6-311++G(d,p)/DMSO				
**Atoms numbering**	**I**	**δexp**	**Δ**	**Δδ**
**H1**	7.22	7.21	-0.01	0%
**H2**	7.78	7.41	-0.37	5%
**H3**	7.74	7.36	-0.38	5%
**H4**	7.79	7.41	-0.38	5%
**H5**	7.68	7.21	-0.47	6%
**H6**	6.78	6.93	0.15	2%
**H7**	7.45	7.69	0.24	3%
**H8**	7.84	7.21	-0.63	8%
**H9**	7.99	7.21	-0.78	10%
**H10**	7.99	7.69	-0.3	4%
**H11**	7.92	7.67	-0.25	3%
**H12**	7.86	7.47	-0.39	5%
**H13**	7.77	7.36	-0.41	5%
**H14**	7.86	7.47	-0.39	5%
**H15**	7.93	7.67	-0.26	3%
**H16**	7.37	7.69	0.32	4%
**H17**	7.4	6.98	-0.42	6%
**H18**	7.37	7.15	-0.22	3%

CAM-B3LYP/6-31G(d,p)/DMSO				
**Atoms numbering**	I	δexp	Δ	Δδ
**H1**	6.93	7.21	0.28	4%
**H2**	7.66	7.41	-0.25	3%
**H3**	7.63	7.36	-0.27	4%
**H4**	7.68	7.41	-0.27	4%
**H5**	7.56	7.21	-0.35	5%
**H6**	6.54	6.93	0.39	6%
**H7**	7.29	7.69	0.4	5%
**H8**	7.76	7.21	-0.55	7%
**H9**	7.92	7.21	-0.71	9%
**H10**	7.93	7.69	-0.24	3%
**H11**	7.85	7.67	-0.18	2%
**H12**	7.77	7.47	-0.3	4%
**H13**	7.67	7.36	-0.31	4%
**H14**	7.76	7.47	-0.29	4%
**H15**	7.85	7.67	-0.18	2%
**H16**	7.14	7.69	0.55	8%
**H17**	7.23	6.98	-0.25	3%
**H18**	7.22	7.15	-0.07	1%

APF/6-31G(d,p)/DMSO				
**Atoms numbering**	I	δexp	Δ	Δδ
**H1**	6.98	7.21	0.23	3%
**H2**	7.7	7.41	-0.29	4%
**H3**	7.67	7.36	-0.31	4%
**H4**	7.72	7.41	-0.31	4%
**H5**	7.6	7.21	-0.39	5%
**H6**	6.69	6.93	0.24	4%
**H7**	7.31	7.69	0.38	5%
**H8**	7.8	7.21	-0.59	8%
**H9**	7.96	7.21	-0.75	9%
**H10**	7.96	7.69	-0.27	3%
**H11**	7.9	7.67	-0.23	3%
**H12**	7.81	7.47	-0.34	4%
**H13**	7.71	7.36	-0.35	5%
**H14**	7.8	7.47	-0.33	4%
**H15**	7.9	7.67	-0.23	3%
**H16**	7.15	7.69	0.54	8%
**H17**	7.28	6.98	-0.3	4%
**H18**	7.24	7.15	-0.09	1%

M06L/6-31G(d,p)/DMSO				
**Atoms numbering**	I	δexp	Δ	Δδ
**H1**	6.49	7.21	0.72	11%
**H2**	7.55	7.41	-0.14	2%
**H3**	7.54	7.36	-0.18	2%
**H4**	7.59	7.41	-0.18	2%
**H5**	7.4	7.21	-0.19	3%
**H6**	6.68	6.93	0.25	4%
**H7**	7.02	7.69	0.67	10%
**H8**	7.72	7.21	-0.51	7%
**H9**	7.89	7.21	-0.68	9%
**H10**	7.8	7.69	-0.11	1%
**H11**	7.79	7.67	-0.12	2%
**H12**	7.62	7.47	-0.15	2%
**H13**	7.43	7.36	-0.07	1%
**H14**	7.67	7.47	-0.2	3%
**H15**	7.83	7.67	-0.16	2%
**H16**	7.22	7.69	0.47	6%
**H17**	7.34	6.98	-0.36	5%
**H18**	7.32	7.15	-0.17	2%

PBE1PBE/6-31G(d,p)/DMSO				
**Atoms numbering**	I	δexp	Δ	Δδ
**H1**	6.95	7.21	0.26	4%
**H2**	7.71	7.41	-0.3	4%
**H3**	7.69	7.36	-0.33	4%
**H4**	7.74	7.41	-0.33	4%
**H5**	7.62	7.21	-0.41	5%
**H6**	6.69	6.93	0.24	4%
**H7**	7.31	7.69	0.38	5%
**H8**	7.82	7.21	-0.61	8%
**H9**	7.99	7.21	-0.78	10%
**H10**	7.98	7.69	-0.29	4%
**H11**	7.92	7.67	-0.25	3%
**H12**	7.83	7.47	-0.36	5%
**H13**	7.73	7.36	-0.37	5%
**H14**	7.82	7.47	-0.35	4%
**H15**	7.92	7.67	-0.25	3%
**H16**	7.13	7.69	0.56	8%
**H17**	7.29	6.98	-0.31	4%
**H18**	7.25	7.15	-0.1	1%

M062X/6-31G(d,p)/DMSO				
**Atoms numbering**	**I**	**δexp**	**Δ**	**Δδ**
**H1**	7.16	7.21	0.05	1%
**H2**	8.31	7.41	-0.9	11%
**H3**	8.32	7.36	-0.96	12%
**H4**	8.32	7.41	-0.91	11%
**H5**	8.18	7.21	-0.97	12%
**H6**	6.91	6.93	0.02	0%
**H7**	7.66	7.69	0.03	0%
**H8**	8.38	7.21	-1.17	14%
**H9**	8.7	7.21	-1.49	17%
**H10**	8.67	7.69	-0.98	11%
**H11**	8.54	7.67	-0.87	10%
**H12**	8.41	7.47	-0.94	11%
**H13**	8.35	7.36	-0.99	12%
**H14**	8.5	7.47	-1.03	12%
**H15**	8.63	7.67	-0.96	11%
**H16**	7.35	7.69	0.34	5%
**H17**	7.83	6.98	-0.85	11%
**H18**	7.78	7.15	-0.63	8%

wB97XD/6-31G(d,p)/DMSO				
**Atoms numbering**	**I**	**δexp**	**Δ**	**Δδ**
**H1**	6.8	7.21	0.41	6%
**H2**	7.7	7.41	-0.29	4%
**H3**	7.71	7.36	-0.35	5%
**H4**	7.75	7.41	-0.34	4%
**H5**	7.63	7.21	-0.42	5%
**H6**	6.61	6.93	0.32	5%
**H7**	7.26	7.69	0.43	6%
**H8**	7.77	7.21	-0.56	7%
**H9**	7.95	7.21	-0.74	9%
**H10**	8.03	7.69	-0.34	4%
**H11**	7.9	7.67	-0.23	3%
**H12**	7.85	7.47	-0.38	5%
**H13**	7.76	7.36	-0.4	5%
**H14**	7.83	7.47	-0.36	5%
**H15**	7.88	7.67	-0.21	3%
**H16**	7.11	7.69	0.58	8%
**H17**	7.31	6.98	-0.33	5%
**H18**	7.28	7.15	-0.13	2%

Figure S1. Cartesian coordinates for isoconazole **1** optimized in methanol (CPCM solvation model) at the B3LYP/6-311++G(2d,3p)//B3LYP/6-31G(d,p) level of theory (charge = 0, multiplicity = 1).

Cl	11.695354000	-0.028880000	-3.819573000
Cl	12.168725000	-3.548224000	-7.901215000
Cl	6.095608000	3.235872000	-3.938507000
Cl	6.033791000	-0.301662000	-8.152558000
O	7.343537000	0.003741000	-4.798272000
N	5.718586000	0.406005000	-0.877209000
N	7.486035000	-0.431188000	-1.948940000
C	6.124774000	-0.383115000	-1.854100000
H	5.490170000	-0.946477000	-2.523867000
C	7.980363000	0.400412000	-0.961798000
H	9.040231000	0.540232000	-0.813828000
C	6.878898000	0.902004000	-0.315493000
H	6.856864000	1.581971000	0.525262000
C	8.261022000	-1.157477000	-2.943810000
H	9.188836000	-1.502087000	-2.482512000
H	7.690906000	-2.037214000	-3.252354000
C	8.587935000	-0.312522000	-4.189831000
H	9.086903000	0.610973000	-3.868828000
C	9.510396000	-1.091785000	-5.120781000
C	10.907655000	-1.044195000	-5.030589000
C	11.734219000	-1.787225000	-5.872778000
H	12.811348000	-1.726083000	-5.781746000
C	11.139798000	-2.605887000	-6.829516000
C	9.754734000	-2.690993000	-6.952597000
H	9.306112000	-3.332631000	-7.701726000
C	8.959873000	-1.934406000	-6.095446000
H	7.880152000	-1.978185000	-6.185626000
C	7.410856000	1.086790000	-5.737749000
H	7.955248000	1.927183000	-5.294706000
H	7.942654000	0.775407000	-6.641793000
C	6.002952000	1.506245000	-6.077022000
C	5.313588000	2.474331000	-5.324752000
C	4.012804000	2.875670000	-5.620178000
H	3.527802000	3.629815000	-5.012080000
C	3.352446000	2.293263000	-6.699484000
H	2.339209000	2.597075000	-6.940120000
C	3.985612000	1.320925000	-7.469583000
H	3.479449000	0.858152000	-8.308229000
C	5.287552000	0.943170000	-7.148063000

Figure S2. Cartesian coordinates for isoconazole **1** optimized in methanol (CPCM solvation model) at the wB97XD/6-311++G(2d,3p)//wB97XD/6-31G(d,p) level of theory (charge = 0, multiplicity = 1).

N	1.082446000	0.658627000	2.488690000
N	1.719071000	-1.459340000	2.279079000
C	-0.961355000	2.040482000	2.672411000
C	-1.756837000	0.953610000	2.314634000
C	-3.105703000	1.138426000	2.016009000
C	-3.668064000	2.409760000	2.071189000
C	-2.875287000	3.500198000	2.426267000
C	-1.530189000	3.315086000	2.724473000
C	0.504487000	1.883479000	3.048321000
C	0.750921000	1.944710000	4.548858000
C	-0.199247000	1.506663000	5.470537000
C	0.075246000	1.532069000	6.833231000
C	1.304240000	1.998558000	7.311466000
C	2.252088000	2.441072000	6.380661000
C	1.978197000	2.413719000	5.019782000
C	1.594920000	2.025854000	8.767987000
C	2.287644000	3.099576000	9.340145000
C	2.560128000	3.123622000	10.704301000
C	2.143966000	2.074508000	11.520488000
C	1.453798000	1.001001000	10.962575000
C	1.182226000	0.976799000	9.598342000
C	1.276744000	-0.537081000	3.108024000
C	1.806029000	-0.828570000	1.059485000
C	1.414010000	0.477089000	1.166440000
H	-1.324485000	-0.040950000	2.263811000
H	-3.715244000	0.284681000	1.737948000
H	-4.717721000	2.552725000	1.836017000
H	-3.304641000	4.496013000	2.467246000
H	-0.914682000	4.166237000	3.003470000
H	1.058608000	2.709019000	2.590141000
H	-1.164761000	1.151387000	5.124891000
H	-0.685674000	1.204799000	7.534842000
H	3.219868000	2.793530000	6.723548000
H	2.731077000	2.751629000	4.313199000
H	2.597714000	3.932318000	8.715906000
H	3.092509000	3.967578000	11.131222000
H	2.356283000	2.093282000	12.584526000
H	1.131902000	0.175499000	11.589373000
H	0.662411000	0.125417000	9.169199000
H	1.088467000	-0.671726000	4.163018000
H	2.152193000	-1.345420000	0.175497000
H	1.337703000	1.278066000	0.447750000

Figure S3. Vertical excited states and their parameters computed for isoconazole **1** in methanol.

Excitation energies: APF (E/au, dE/eV, L/nm)

E0= -2719.0103862

E1= -2718.8311010 dE1= 4.8786 L1= 254.14 f1= 0.0121

E2= -2718.8276943 dE2= 4.9713 L2= 249.40 f2= 0.0076

E3= -2718.8273673 dE3= 4.9802 L3= 248.95 f3= 0.0196

E4= -2718.8236262 dE4= 5.0820 L4= 243.97 f4= 0.0221

E5= -2718.8221525 dE5= 5.1221 L5= 242.06 f5= 0.0003

E6= -2718.8215903 dE6= 5.1374 L6= 241.34 f6= 0.0067

E7= -2718.8112637 dE7= 5.4184 L7= 228.82 f7= 0.1567

E8= -2718.8053397 dE8= 5.5796 L8= 222.21 f8= 0.0908

E9= -2718.8038661 dE9= 5.6197 L9= 220.62 f9= 0.0145

E10= -2718.8023777 dE10= 5.6602 L10= 219.05 f10= 0.0034

Excitation energies: B3LYP/6311++G(d,p) (E/au, dE/eV, L/nm)

E0= -2720.2308458

E1= -2720.0568599 dE1= 4.7344 L1= 261.88 f1= 0.0084

E2= -2720.0511821 dE2= 4.8889 L2= 253.60 f2= 0.0095

E3= -2720.0500833 dE3= 4.9188 L3= 252.06 f3= 0.0163

E4= -2720.0485840 dE4= 4.9596 L4= 249.99 f4= 0.0119

E5= -2720.0477791 dE5= 4.9815 L5= 248.89 f5= 0.0007

E6= -2720.0465738 dE6= 5.0143 L6= 247.26 f6= 0.0075

E7= -2720.0363575 dE7= 5.2923 L7= 234.27 f7= 0.1221

E8= -2720.0287393 dE8= 5.4996 L8= 225.44 f8= 0.0495

E9= -2720.0285997 dE9= 5.5034 L9= 225.29 f9= 0.0659

E10= -2720.0281697 dE10= 5.5151 L10= 224.81 f10= 0.0140

Excitation energies: CAM-B3LYP (E/au, dE/eV, L/nm)

E0= -2719.7708386

E1= -2719.5814474 dE1= 5.1536 L1= 240.58 f1= 0.0097

E2= -2719.5809035 dE2= 5.1684 L2= 239.89 f2= 0.0065

E3= -2719.5617938 dE3= 5.6884 L3= 217.96 f3= 0.1618

E4= -2719.5600446 dE4= 5.7360 L4= 216.15 f4= 0.0574

E5= -2719.5479871 dE5= 6.0641 L5= 204.46 f5= 0.0523

E6= -2719.5434817 dE6= 6.1867 L6= 200.41 f6= 0.0040

E7= -2719.5424968 dE7= 6.2135 L7= 199.54 f7= 0.0365

E8= -2719.5419676 dE8= 6.2279 L8= 199.08 f8= 0.0979

E9= -2719.5403543 dE9= 6.2718 L9= 197.69 f9= 0.5574

E10= -2719.5387006 dE10= 6.3168 L10= 196.28 f10= 0.6162

Excitation energies: M062X (E/au, dE/eV, L/nm)

E0= -2719.7906146

E1= -2719.5999371 dE1= 5.1886 L1= 238.95 f1= 0.0091

E2= -2719.5996835 dE2= 5.1955 L2= 238.64 f2= 0.0122

E3= -2719.5785012 dE3= 5.7719 L3= 214.81 f3= 0.2749

E4= -2719.5766307 dE4= 5.8228 L4= 212.93 f4= 0.0549

E5= -2719.5726691 dE5= 5.9306 L5= 209.06 f5= 0.0194

E6= -2719.5672045 dE6= 6.0793 L6= 203.94 f6= 0.0125

E7= -2719.5647386 dE7= 6.1464 L7= 201.72 f7= 0.0646

E8= -2719.5629600 dE8= 6.1948 L8= 200.14 f8= 0.0170

E9= -2719.5625998 dE9= 6.2046 L9= 199.83 f9= 0.0019

E10= -2719.5608616 dE10= 6.2519 L10= 198.31 f10= 0.1109

Excitation energies: M06L (E/au, dE/eV, L/nm)

E0= -2720.0234887

E1= -2719.8737389 dE1= 4.0749 L1= 304.26 f1= 0.0054

E2= -2719.8692298 dE2= 4.1976 L2= 295.37 f2= 0.0039

E3= -2719.8665728 dE3= 4.2699 L3= 290.37 f3= 0.0086

E4= -2719.8661979 dE4= 4.2801 L4= 289.67 f4= 0.0039

E5= -2719.8529461 dE5= 4.6407 L5= 267.17 f5= 0.0207

E6= -2719.8483672 dE6= 4.7653 L6= 260.18 f6= 0.0057

E7= -2719.8476689 dE7= 4.7843 L7= 259.15 f7= 0.0012

E8= -2719.8463864 dE8= 4.8192 L8= 257.27 f8= 0.0006

E9= -2719.8450928 dE9= 4.8544 L9= 255.40 f9= 0.0105

E10= -2719.8431929 dE10= 4.9061 L10= 252.72 f10= 0.0089

Excitation energies: B3LYP (E/au, dE/eV, L/nm)

E0= -2720.2302318

E1= -2720.0574071 dE1= 4.7028 L1= 263.64 f1= 0.0102

E2= -2720.0517404 dE2= 4.8570 L2= 255.27 f2= 0.0097

E3= -2720.0500095 dE3= 4.9041 L3= 252.82 f3= 0.0153

E4= -2720.0495060 dE4= 4.9178 L4= 252.11 f4= 0.0144

E5= -2720.0487527 dE5= 4.9383 L5= 251.07 f5= 0.0004

E6= -2720.0468417 dE6= 4.9903 L6= 248.45 f6= 0.0064

E7= -2720.0361256 dE7= 5.2819 L7= 234.74 f7= 0.1192

E8= -2720.0290073 dE8= 5.4756 L8= 226.43 f8= 0.0892

E9= -2720.0287096 dE9= 5.4837 L9= 226.10 f9= 0.0029

E10= -2720.0285589 dE10= 5.4878 L10= 225.93 f10= 0.0384

Excitation energies: PBE1PBE (E/au, dE/eV, L/nm)

E0= -2718.5507096

E1= -2718.3675914 dE1= 4.9829 L1= 248.82 f1= 0.0107

E2= -2718.3664926 dE2= 5.0128 L2= 247.33 f2= 0.0009

E3= -2718.3664044 dE3= 5.0152 L3= 247.22 f3= 0.0292

E4= -2718.3603151 dE4= 5.1809 L4= 239.31 f4= 0.0290

E5= -2718.3586907 dE5= 5.2251 L5= 237.29 f5= 0.0005

E6= -2718.3583674 dE6= 5.2339 L6= 236.89 f6= 0.0082

E7= -2718.3491359 dE7= 5.4851 L7= 226.04 f7= 0.1677

E8= -2718.3445533 dE8= 5.6098 L8= 221.01 f8= 0.0753

E9= -2718.3415619 dE9= 5.6912 L9= 217.85 f9= 0.0110

E10= -2718.3388535 dE10= 5.7649 L10= 215.07 f10= 0.0039

Excitation energies: wB97XD (E/au, dE/eV, L/nm)

E0= -2719.8771347

E1= -2719.6885630 dE1= 5.1313 L1= 241.62 f1= 0.0089

E2= -2719.6870747 dE2= 5.1718 L2= 239.73 f2= 0.0053

E3= -2719.6673513 dE3= 5.7085 L3= 217.19 f3= 0.0884

E4= -2719.6667302 dE4= 5.7254 L4= 216.55 f4= 0.0764

E5= -2719.6664951 dE5= 5.7318 L5= 216.31 f5= 0.0238

E6= -2719.6608136 dE6= 5.8864 L6= 210.63 f6= 0.0024

E7= -2719.6502004 dE7= 6.1752 L7= 200.78 f7= 0.0160

E8= -2719.6486753 dE8= 6.2167 L8= 199.44 f8= 0.0463

E9= -2719.6471576 dE9= 6.2580 L9= 198.12 f9= 1.0109

E10= -2719.6461396 dE10= 6.2857 L10= 197.25 f10= 0.0048

Figure S4. Vertical excited states and their parameters computed for isoconazole **2** in methanol.

Excitation energies: APF (E/au, dE/eV, L/nm)

E0= -958.1365016

E1= -957.9659591 dE1= 4.6407 L1= 267.17 f1= 0.6865

E2= -957.9626774 dE2= 4.7300 L2= 262.12 f2= 0.1075

E3= -957.9575288 dE3= 4.8701 L3= 254.58 f3= 0.0202

E4= -957.9537069 dE4= 4.9741 L4= 249.26 f4= 0.0038

E5= -957.9455228 dE5= 5.1968 L5= 238.58 f5= 0.0049

E6= -957.9434943 dE6= 5.2520 L6= 236.07 f6= 0.0524

E7= -957.9410063 dE7= 5.3197 L7= 233.06 f7= 0.0002

E8= -957.9404955 dE8= 5.3336 L8= 232.46 f8= 0.0014

E9= -957.9362730 dE9= 5.4485 L9= 227.56 f9= 0.0098

E10= -957.9352000 dE10= 5.4777 L10= 226.34 f10= 0.0205

Excitation energies: B3LYP/6-311++G(d,p) (E/au, dE/eV, L/nm)

E0= -958.9688780

E1= -958.8011063 dE1= 4.5653 L1= 271.58 f1= 0.5439

E2= -958.7983354 dE2= 4.6407 L2= 267.17 f2= 0.2132

E3= -958.7925915 dE3= 4.7970 L3= 258.46 f3= 0.0212

E4= -958.7892143 dE4= 4.8889 L4= 253.60 f4= 0.0035

E5= -958.7813279 dE5= 5.1035 L5= 242.94 f5= 0.0045

E6= -958.7801519 dE6= 5.1355 L6= 241.43 f6= 0.0465

E7= -958.7783659 dE7= 5.1841 L7= 239.16 f7= 0.0012

E8= -958.7774876 dE8= 5.2080 L8= 238.07 f8= 0.0009

E9= -958.7738053 dE9= 5.3082 L9= 233.57 f9= 0.0127

E10= -958.7728829 dE10= 5.3333 L10= 232.47 f10= 0.0149

Excitation energies: B3LYP (E/au, dE/eV, L/nm)

E0= -958.9684271

E1= -958.8020961 dE1= 4.5261 L1= 273.93 f1= 0.5857

E2= -958.7991965 dE2= 4.6050 L2= 269.24 f2= 0.1844

E3= -958.7935041 dE3= 4.7599 L3= 260.48 f3= 0.0170

E4= -958.7895462 dE4= 4.8676 L4= 254.71 f4= 0.0038

E5= -958.7817112 dE5= 5.0808 L5= 244.03 f5= 0.0055

E6= -958.7805389 dE6= 5.1127 L6= 242.50 f6= 0.0446

E7= -958.7787860 dE7= 5.1604 L7= 240.26 f7= 0.0022

E8= -958.7780510 dE8= 5.1804 L8= 239.33 f8= 0.0010

E9= -958.7742805 dE9= 5.2830 L9= 234.69 f9= 0.0123

E10= -958.7734610 dE10= 5.3053 L10= 233.70 f10= 0.0156

Excitation energies: CAM-B3LYP (E/au, dE/eV, L/nm)

E0= -958.4286438

E1= -958.2453677 dE1= 4.9872 L1= 248.61 f1= 0.8157

E2= -958.2410570 dE2= 5.1045 L2= 242.89 f2= 0.0056

E3= -958.2374702 dE3= 5.2021 L3= 238.34 f3= 0.0004

E4= -958.2307929 dE4= 5.3838 L4= 230.29 f4= 0.0031

E5= -958.2117788 dE5= 5.9012 L5= 210.10 f5= 0.0188

E6= -958.2089381 dE6= 5.9785 L6= 207.38 f6= 0.0635

E7= -958.2054873 dE7= 6.0724 L7= 204.18 f7= 0.0310

E8= -958.2051823 dE8= 6.0807 L8= 203.90 f8= 0.0319

E9= -958.2040908 dE9= 6.1104 L9= 202.91 f9= 0.0240

E10= -958.2021321 dE10= 6.1637 L10= 201.15 f10= 0.0630

Excitation energies: M062X (E/au, dE/eV, L/nm)

E0= -958.5837906

E1= -958.3994561 dE1= 5.0160 L1= 247.18 f1= 0.8648

E2= -958.3958252 dE2= 5.1148 L2= 242.40 f2= 0.0140

E3= -958.3913382 dE3= 5.2369 L3= 236.75 f3= 0.0003

E4= -958.3844550 dE4= 5.4242 L4= 228.58 f4= 0.0039

E5= -958.3710966 dE5= 5.7877 L5= 214.22 f5= 0.0211

E6= -958.3655953 dE6= 5.9374 L6= 208.82 f6= 0.0197

E7= -958.3637100 dE7= 5.9887 L7= 207.03 f7= 0.0049

E8= -958.3625304 dE8= 6.0208 L8= 205.93 f8= 0.0131

E9= -958.3618615 dE9= 6.0390 L9= 205.30 f9= 0.0805

E10= -958.3600277 dE10= 6.0889 L10= 203.62 f10= 0.1337

Excitation energies: M06L (E/au, dE/eV, L/nm)

E0= -958.8198448

E1= -958.6747034 dE1= 3.9495 L1= 313.92 f1= 0.0056

E2= -958.6614186 dE2= 4.3110 L2= 287.60 f2= 0.5435

E3= -958.6555607 dE3= 4.4704 L3= 277.35 f3= 0.0291

E4= -958.6535028 dE4= 4.5264 L4= 273.91 f4= 0.0148

E5= -958.6513676 dE5= 4.5845 L5= 270.44 f5= 0.1341

E6= -958.6494714 dE6= 4.6361 L6= 267.43 f6= 0.0166

E7= -958.6479573 dE7= 4.6773 L7= 265.08 f7= 0.0240

E8= -958.6475677 dE8= 4.6879 L8= 264.47 f8= 0.0023

E9= -958.6460132 dE9= 4.7302 L9= 262.11 f9= 0.0171

E10= -958.6449071 dE10= 4.7603 L10= 260.46 f10= 0.0048

Excitation energies: PBE1PBE (E/au, dE/eV, L/nm)

E0= -957.8235405

E1= -957.6515390 dE1= 4.6804 L1= 264.90 f1= 0.7678

E2= -957.6470666 dE2= 4.8021 L2= 258.19 f2= 0.0323

E3= -957.6430830 dE3= 4.9105 L3= 252.49 f3= 0.0212

E4= -957.6393272 dE4= 5.0127 L4= 247.34 f4= 0.0036

E5= -957.6308455 dE5= 5.2435 L5= 236.45 f5= 0.0045

E6= -957.6277034 dE6= 5.3290 L6= 232.66 f6= 0.0551

E7= -957.6253331 dE7= 5.3935 L7= 229.88 f7= 0.0002

E8= -957.6246312 dE8= 5.4126 L8= 229.07 f8= 0.0019

E9= -957.6205924 dE9= 5.5225 L9= 224.51 f9= 0.0081

E10= -957.6194275 dE10= 5.5542 L10= 223.23 f10= 0.0257

Excitation energies: wB97XD (E/au, dE/eV, L/nm)

E0= -958.6356254

E1= -958.4491962 dE1= 5.0730 L1= 244.40 f1= 0.8089

E2= -958.4466605 dE2= 5.1420 L2= 241.12 f2= 0.0186

E3= -958.4436397 dE3= 5.2242 L3= 237.33 f3= 0.0003

E4= -958.4376826 dE4= 5.3863 L4= 230.19 f4= 0.0046

E5= -958.4167870 dE5= 5.9549 L5= 208.20 f5= 0.0239

E6= -958.4121382 dE6= 6.0814 L6= 203.88 f6= 0.0755

E7= -958.4098928 dE7= 6.1425 L7= 201.85 f7= 0.0433

E8= -958.4067801 dE8= 6.2272 L8= 199.10 f8= 0.0236

E9= -958.4055674 dE9= 6.2602 L9= 198.05 f9= 0.1753

E10= -958.4043620 dE10= 6.2930 L10= 197.02 f10= 0.3900

Figure S5a. The ^{1}HNMR spectrum of isoconazole (**1**) in DMSO-d_6.

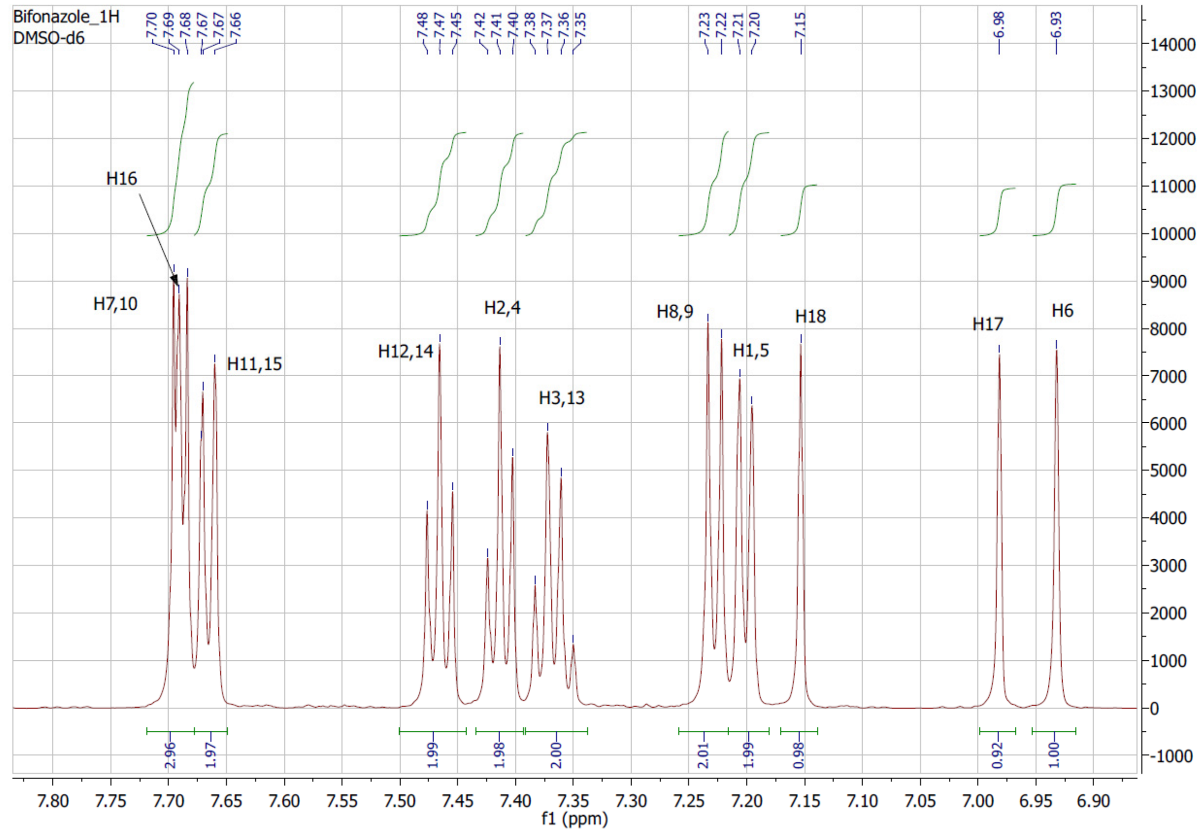

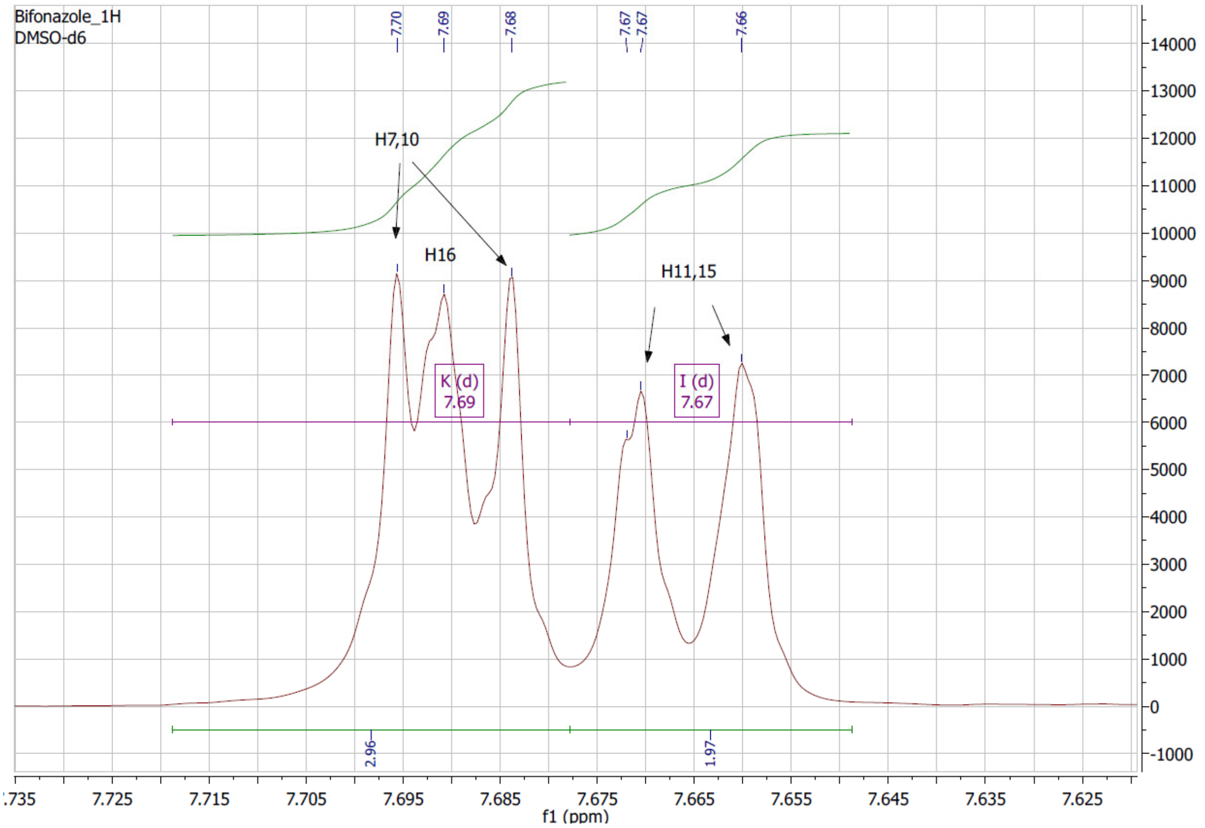

Figure S5b. The NOESY spectrum of isoconazole (**1**) in DMSO-d_6.

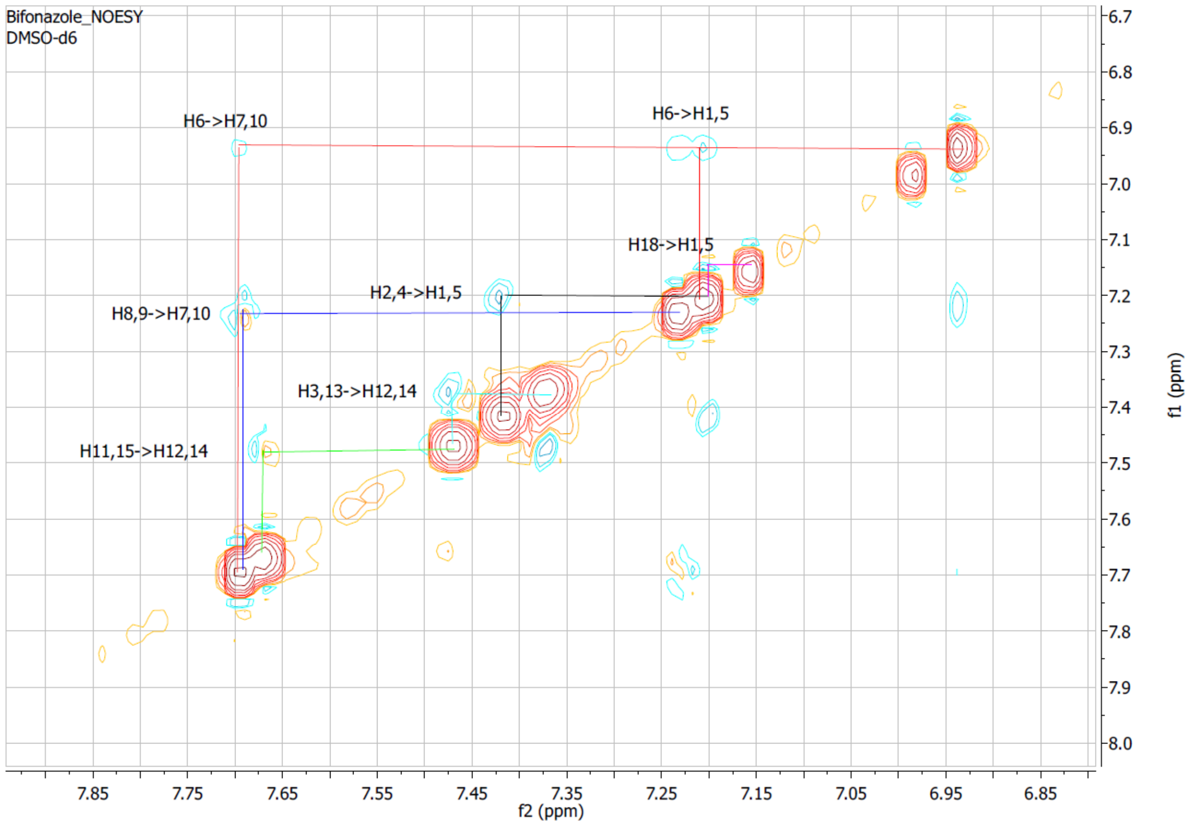

Figure S5c. The HSQC spectrum of isoconazole (**1**) in DMSO-d_6.

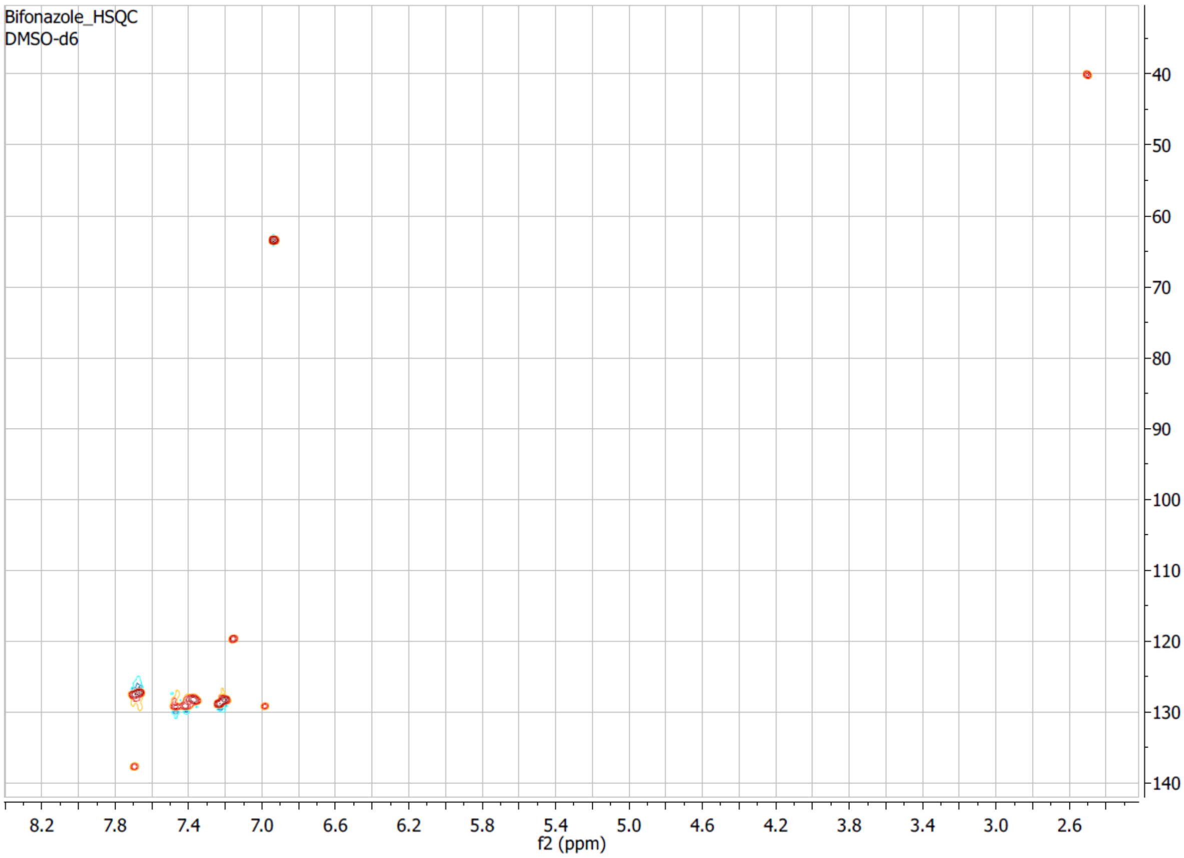

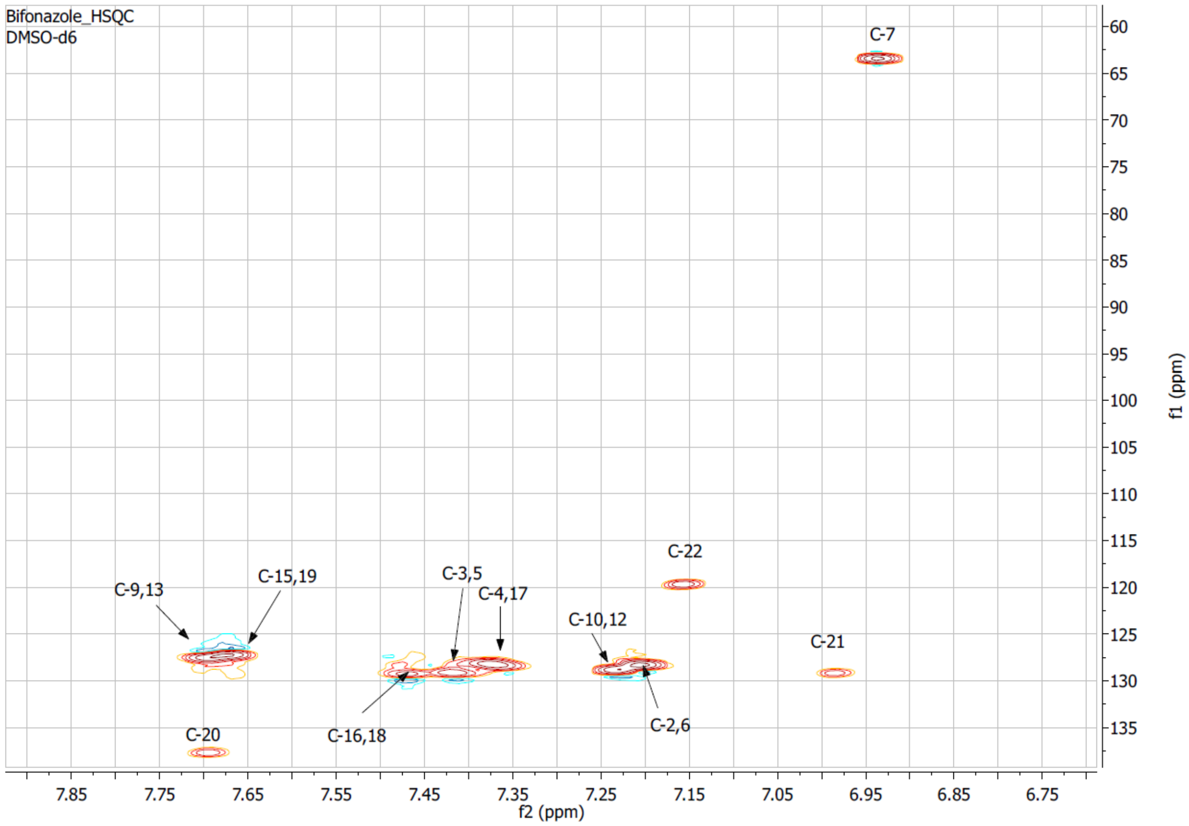

Figure S5d. The HMBC spectrum of isoconazole (**1**) in DMSO-d_6.

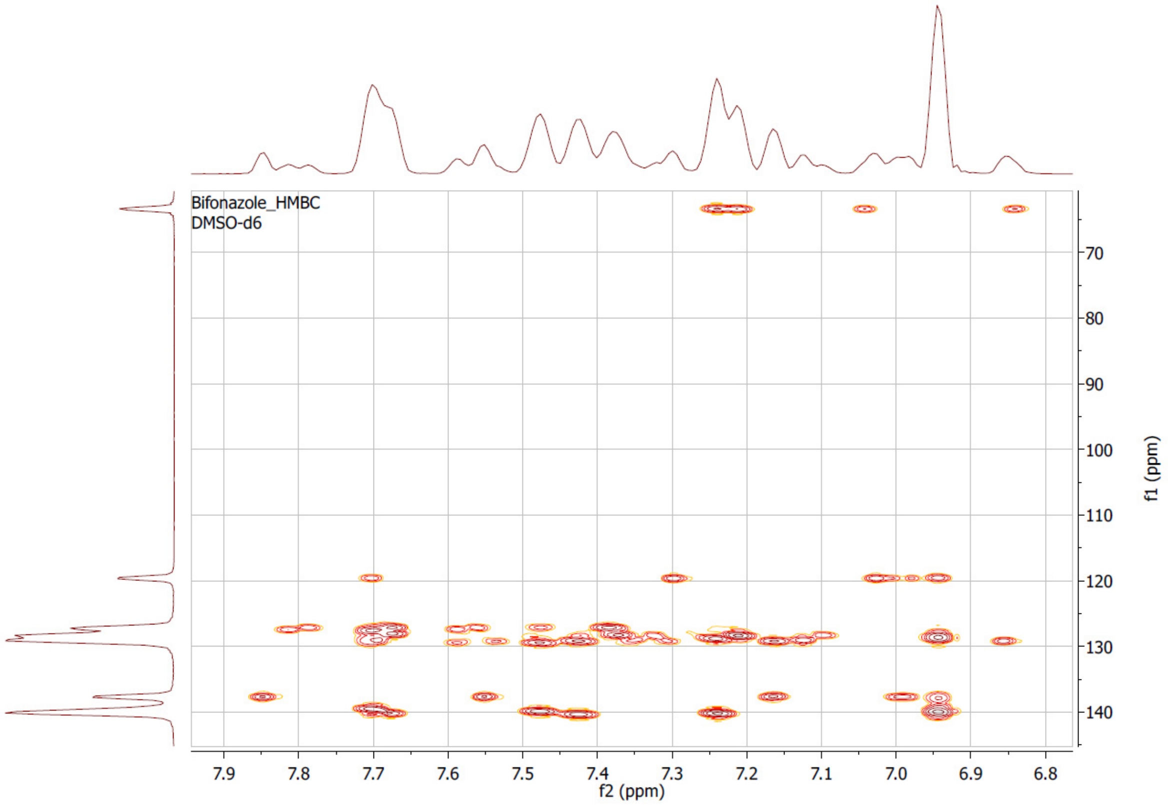

Figure S6a. The ^{1}HNMR spectrum of bifonazole (**2**) in DMSO-d_6.

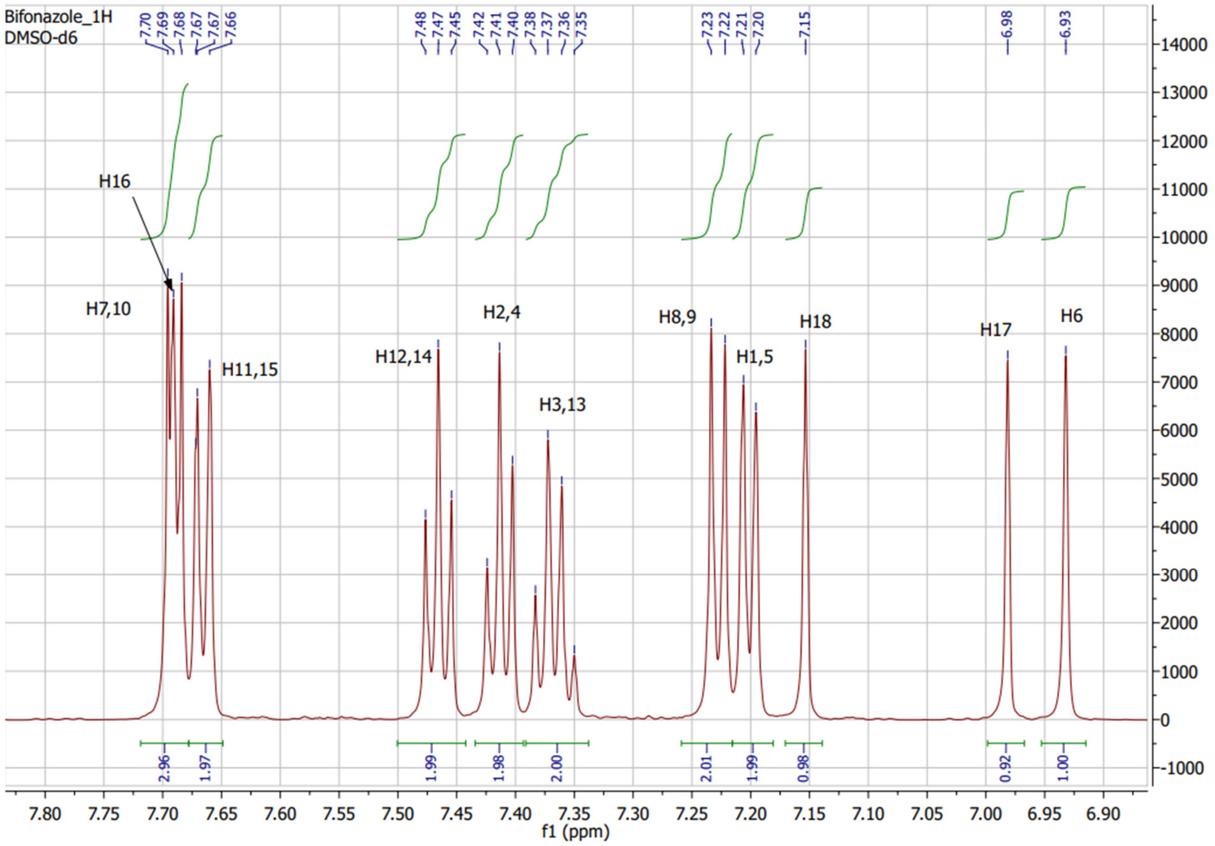

Figure S6b. The NOESY spectrum of bifonazole (**2**) in DMSO-*d*₆.

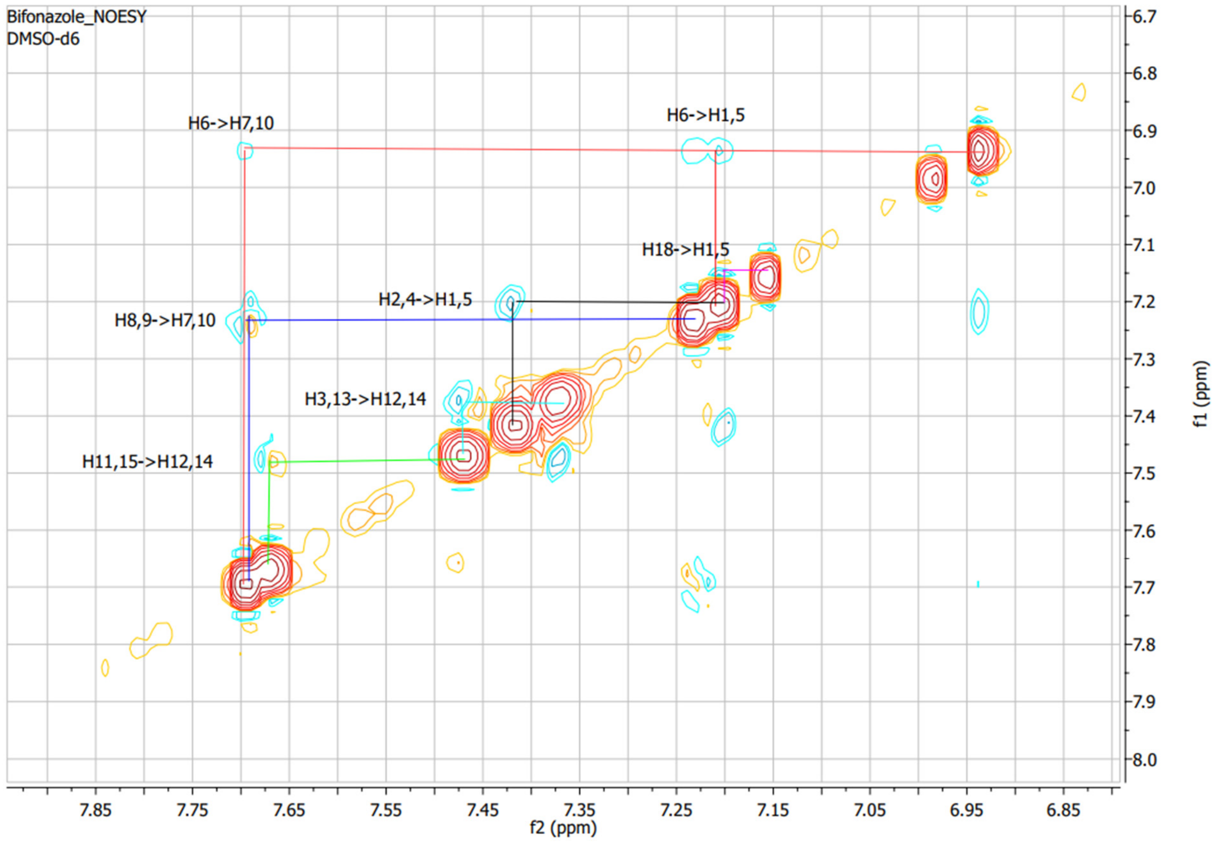

Figure S6c. The HSQC spectrum of bifonazole (**2**) in DMSO-*d*₆.

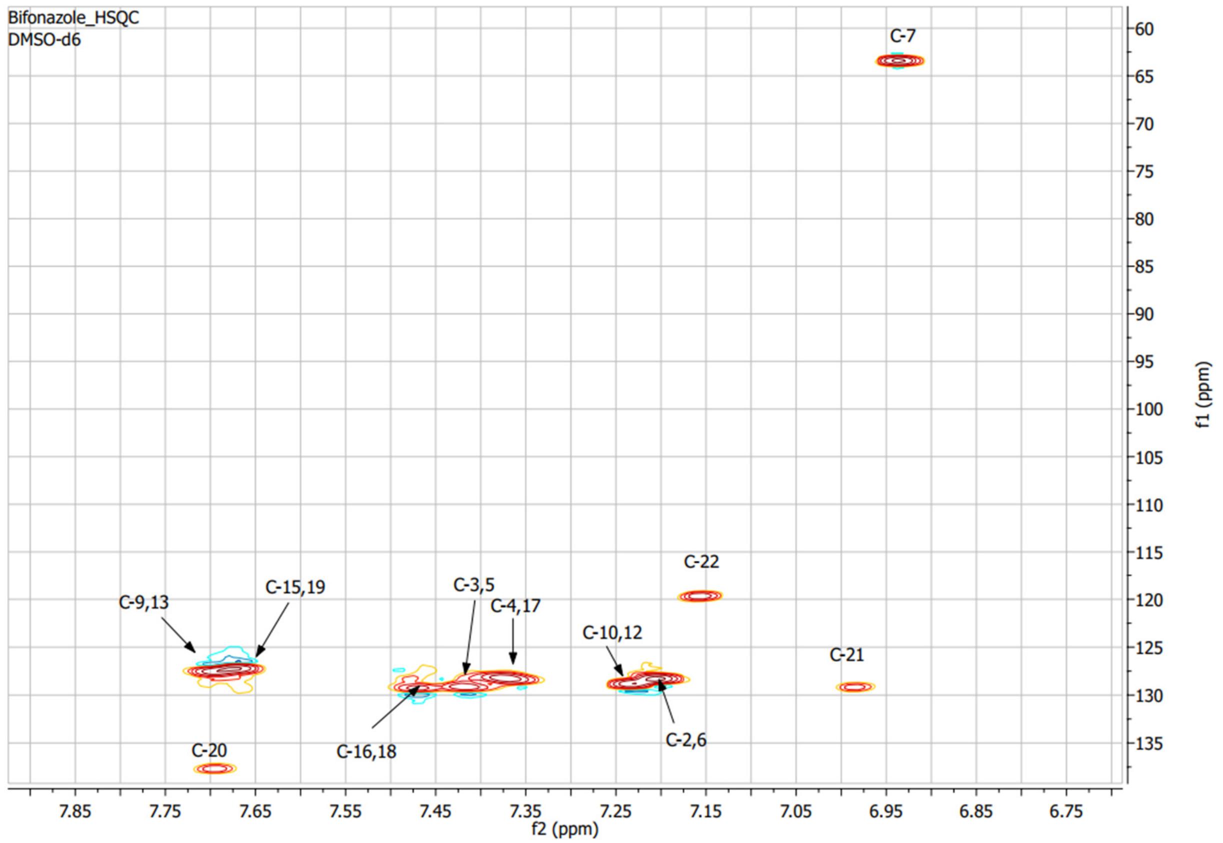

Figure S6d. The HMBC spectrum of bifonazole (**2**) in DMSO-*d*$_6$.

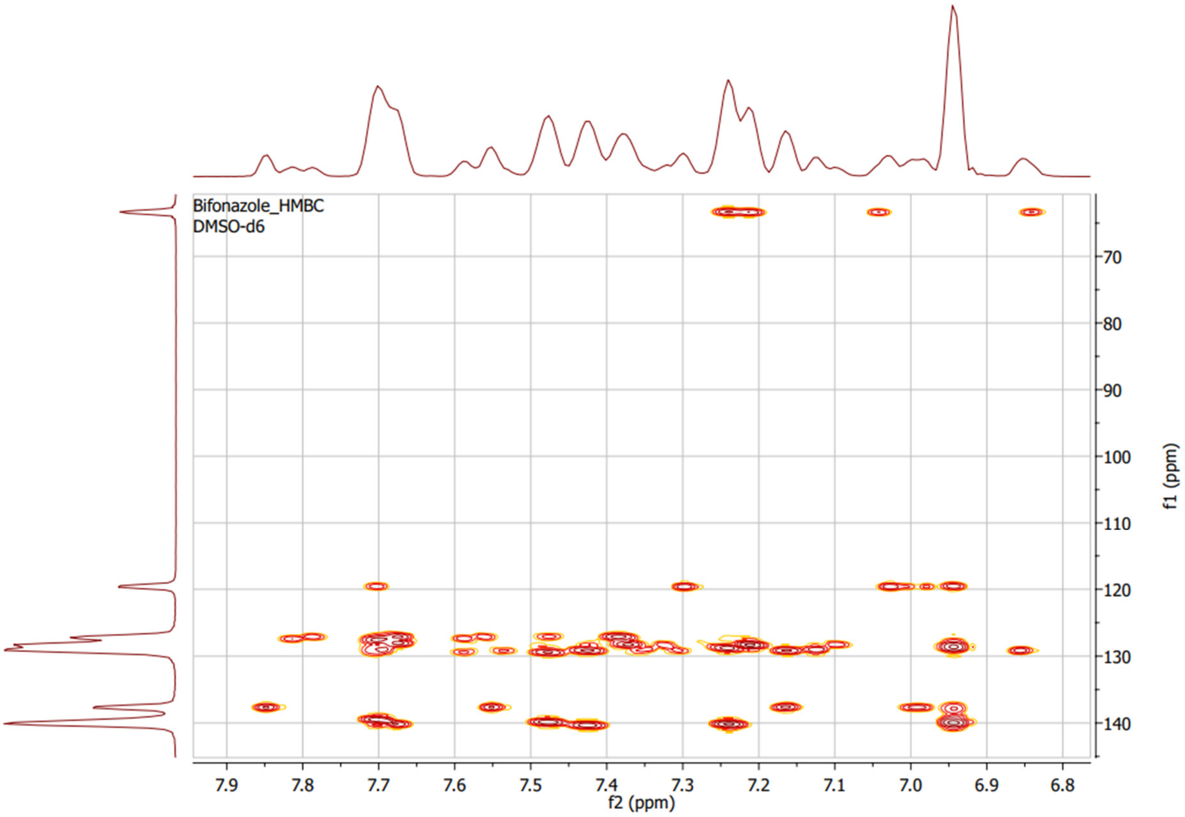

Figure S7. The HR MS spectrum of isoconazole (**1**).

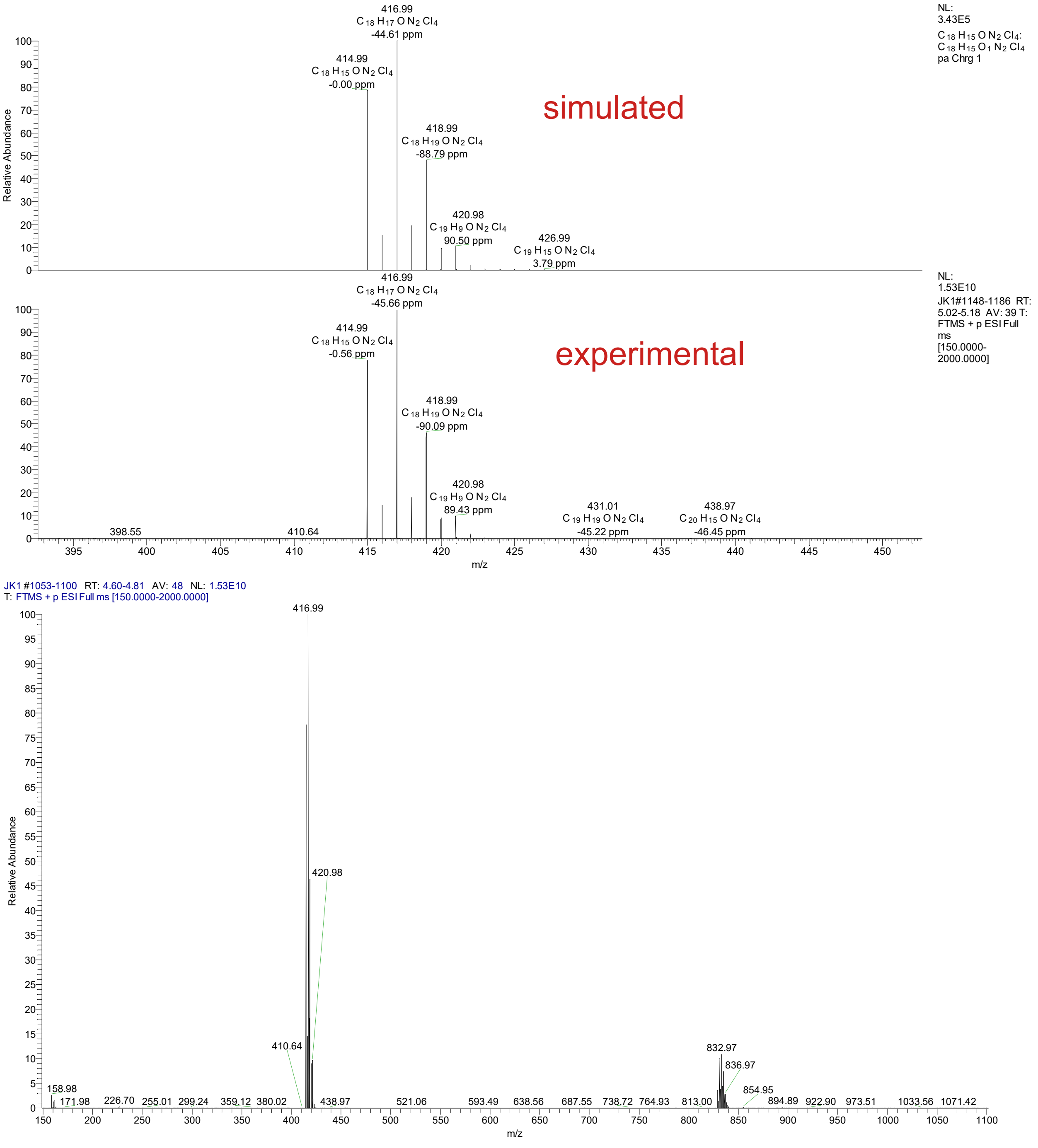

Figure S8. The HR MS spectrum of bifonazole (**2**).

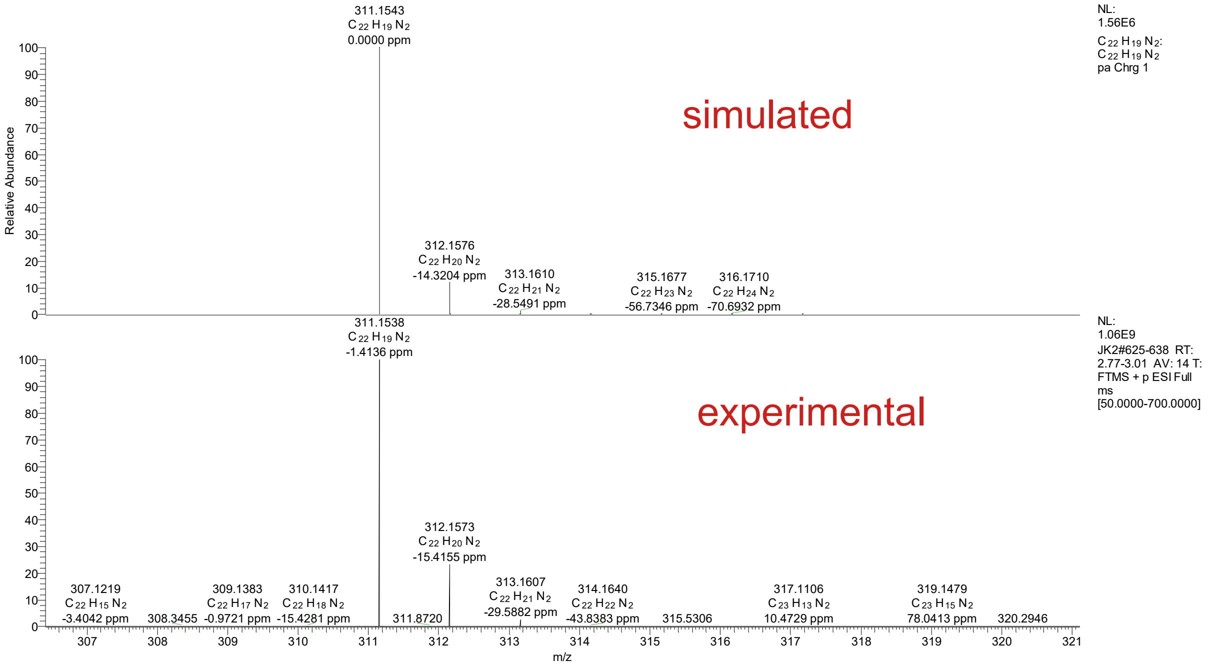

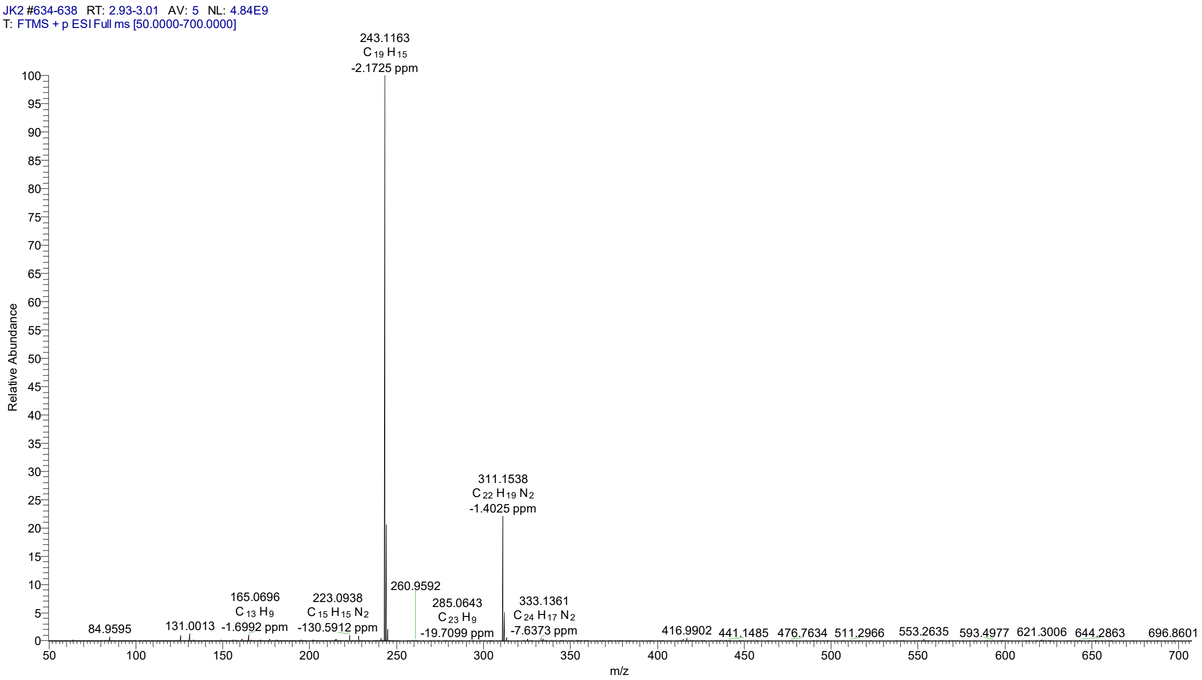

Supplement: Supplementary file 1 [file ijms-24-00520-s001.zip › ijms-2094778-supplementary.pdf]
